# Loss of PKGIβ/IRAG1 Signaling Causes Anemia-Associated Splenomegaly

**DOI:** 10.3390/ijms22115458

**Published:** 2021-05-21

**Authors:** Michael Majer, Sally Prueschenk, Jens Schlossmann

**Affiliations:** Department of Pharmacology and Toxicology, Institute of Pharmacy, University of Regensburg, 93040 Regensburg, Germany; Michael.Majer@chemie.uni-regensburg.de (M.M.); Sally.Prueschenk@chemie.uni-regensburg.de (S.P.)

**Keywords:** anemia, IP_3_R-I, iron deficiency, IRAG, IRAG1, MRVI1, PKGI, PKGIβ, splenomegaly

## Abstract

Inositol 1,4,5-triphosphate receptor-associated cGMP kinase substrate 1 (IRAG1) is a substrate protein of the NO/cGMP-signaling pathway and forms a ternary complex with the cGMP-dependent protein kinase Iβ (PKGIβ) and the inositol triphosphate receptor I (IP_3_R-I). Functional studies about IRAG1 exhibited that IRAG1 is specifically phosphorylated by the PKGIβ, regulating cGMP-mediated IP_3_-dependent Ca^2+^-release. IRAG1 is widely distributed in murine tissues, e.g., in large amounts in smooth muscle-containing tissues and platelets, but also in lower amounts, e.g., in the spleen. The NO/cGMP/PKGI signaling pathway is important in several organ systems. A loss of PKGI causes gastrointestinal disorders, anemia and splenomegaly. Due to the similar tissue distribution of the PKGIβ to IRAG1, we investigated the pathophysiological functions of IRAG1 in this context. Global IRAG1-KO mice developed gastrointestinal bleeding, anemia-associated splenomegaly and iron deficiency. Additionally, *Irag1*-deficiency altered the protein levels of some cGMP/PKGI signaling proteins—particularly a strong decrease in the PKGIβ—in the colon, spleen and stomach but did not change mRNA-expression of the corresponding genes. The present work showed that a loss of IRAG1 and the PKGIβ/IRAG1 signaling has a crucial function in the development of gastrointestinal disorders and anemia-associated splenomegaly. Furthermore, global *Irag1*-deficient mice are possible in vivo model to investigate PKGIβ protein functions.

## 1. Introduction

The inositol 1,4,5-triphosphate receptor-associated cGMP kinase substrate 1 (IRAG1), which is also named IRAG or as human homologue MRVI1, was first described in 2000 [1].

IRAG1 is a substrate protein of the NO/cGMP-signaling pathway. Molecular analysis showed that IRAG1 has a molecular weight of 125 kDa and forms a ternary complex with the cGMP-dependent protein kinase Iβ (PKGIβ) and the inositol triphosphate receptor I (IP_3_R-I) [1]. An investigation of the stability of this complex confirmed that IRAG1 is of great importance for the integrity of this complex [2,3]. IRAG1 is only phosphorylated by PKGIβ and not by PKGIα or PKGII and interacts with the IP_3_R-I [2]. This phosphorylation of IRAG1 and the interaction with the IP_3_R-I lead to a reduction of the cGMP-mediated IP_3_-dependent Ca^2+^ release, which was shown in cellular systems and in the *Irag1^Δ12/Δ12^* mice [2,3,4]. In these mice, exon 12 of IRAG1 is deleted, which is coding for the N-terminus of the coiled-coil domain and resulted in a disrupted in vivo interaction between IRAG1 and IP_3_R-I.

Protein expression of IRAG1 is widespread in murine tissue. Smooth muscle containing tissues and platelets highly expressed IRAG1, while lower amounts of this protein were found for example in the spleen. PKGIβ correlated with IRAG1 in the amount of expression and tissue distribution [5].

Most of the pathophysiological functions of IRAG1 are still unknown. Some physiological functions are the inhibition of NO/cGMP-dependent platelet aggregation [6,7], thrombus formation [6] and regulation of the smooth muscle relaxation [4,8]. In these previous investigations some pathophysiological effects were found. *Irag1^Δ12/Δ12^* mice developed an enlarged gastrointestinal tract and showed a reduced passage time, which demonstrated a stenosis of the pylorus and other functional disorders of gastrointestinal motility [4]. This gastrointestinal phenotype was also found in global *Irag1^−/−^* mice [8] and in both mutant mice (*Irag1^Δ12/Δ12^* and *Irag1^−/−^*) indications of an enlarged spleen were found. In summary, a defect or loss of IRAG1 causes gastrointestinal disorders.

The central part of the NO/cGMP-signaling pathway is related to PKGI. It was reported that a loss of the PKGI in global PKGI-KO mice (genotype: *Prkg1^−/−^*) showed gastrointestinal disorders as enlargement of the gastrointestinal tract and reduced passage time [9]. Further research about the pathophysiological function of the PKGI revealed that *Prkg1^−/−^* mice developed anemia and splenomegaly [10] and gastrointestinal bleedings caused by duodenal ulcers [11]. Treatment with a proton pump inhibitor (PPI) reduced the gastrointestinal bleeding [11,12] and the development of splenomegaly [12].

As already mentioned, IRAG1 and PKGIβ have a similar pattern of protein expression and tissue distribution [5], and IRAG1 and PKGI mouse mutants have similarities in their phenotypes. With the present work, we aimed to investigate what effect a loss of IRAG1 has on the development of anemia and splenomegaly in *Irag1^−/−^* mice.

## 2. Results

### 2.1. Detection of Occult Blood in IRAG1-WT and IRAG1-KO Mice

**Figure 1:**
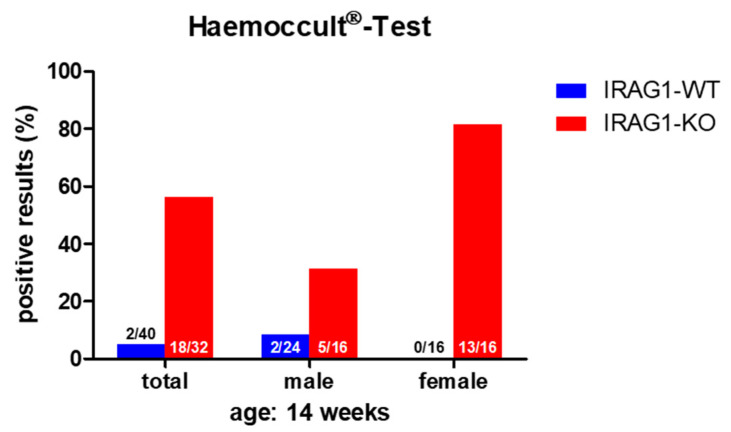
The Haemoccult^®^ Test of IRAG1-WT and IRAG1-KO mice. Occult blood was found in the IRAG1-KO mice, but with a difference in positive results between male and female IRAG1-KO mice. The numbers in the graphs indicated the mice that had a positive Haemoccult^®^ Test compared to the total number of investigated mice; “total” indicates the evaluation of both sexes (male and female) from each genotype.

The proof of blood in feces is an indication of gastrointestinal bleeding. A fast and secure method for the diagnostic of (occult) blood in the feces is the Haemoccult^®^ Test (Section 4.2), a frequently used tool in human diagnostics. As there might be the possibility that *Irag1*-deficient mice may develop gastrointestinal bleeding caused by the similarities of the phenotype to the global *Prkg1*-KO mice [4,8,9], the feces of 14-week-old male and female IRAG1 knockout (KO) mice (IRAG1-KO, genotype: *Irag1^−/−^*) and their wild type (WT)-littermates (IRAG1-WT, genotype: *Irag1^+/+^*) were investigated (Figure 1). In total, 81% of female IRAG1-KO mice had a positive result, whereas no Haemoccult^®^ Tests of female IRAG1-WT mice were positive. In the group of the male mice, 31% of IRAG1-KO mice had occult blood in the feces, and 8% of IRAG1-WT mice showed a positive result. The total result of both sexes presented similar results; in 56% of IRAG1-KO mice and in 5% of IRAG1-WT mice, we detected blood in the feces. To figure out if these findings are age-dependent, we analyzed the feces of six-week-old IRAG1-KO mice and their WT littermates too, and no blood was detected in the feces (Figure S1A).

Therefore, IRAG1-KO mice contained occult blood in the feces, which may be caused by gastrointestinal bleeding.

### 2.2. IRAG1-Deficient Mice Develop Splenomegaly

IRAG1 and PKGIβ expression largely overlap at the protein level [5], and IRAG1 mutant mice have a similar phenotype, e.g., the dilatation of the gastrointestinal tract [4,8], reduced gastrointestinal motility [4] and bleeding (Section 2.1, Figure 1) similar to the global *Prkg1*-KO mice [9], and it is known that *Prkg1*-deficient mice develop splenomegaly [10,12]. To investigate if *Irag1*-deficiency caused an enlarged spleen, the weights of the spleen and body were determined, and the ratio between these weights (spleen/body-ratio) was calculated.

**Figure 2:**
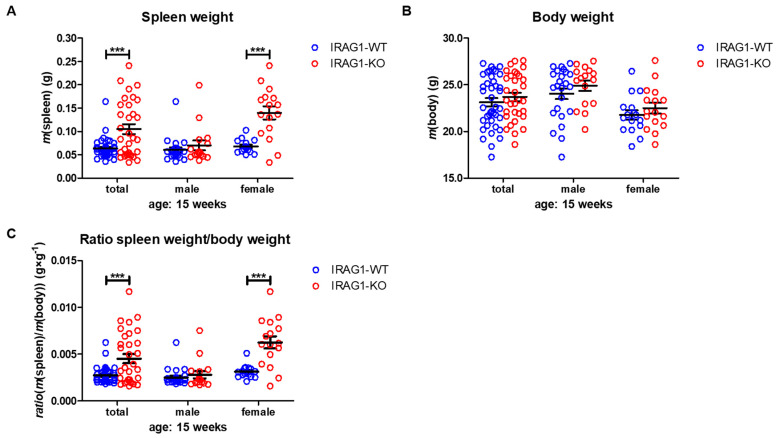
Analysis of spleen and body weight of IRAG1-WT and IRAG1-KO mice. (**A**) Increased spleen weights in IRAG1-KO (total: *n* = 32; female: *n* = 16) mice compared to their WT littermates (total: *n* = 40; female: *n* = 16) were determined, but not in male IRAG1-KO (*n* = 16) and IRAG1-WT mice (*n* = 24). (**B**) No differences in the body weights of IRAG1-KO mice (total: *n* = 32; male: *n* = 16; female: *n* = 16) and their WT littermates (total: *n* = 40; male: *n* = 24; female: *n* = 16) were found. (**C**) Spleen-weight-to-body-weight ratios of IRAG1-KO mice (total: *n* = 32; female: n = 16) were increased compared to their WT littermates (total: *n* = 40; female: *n* = 16), but not in male IRAG1-KO (*n* = 16) and IRAG1-WT mice (*n* = 24). Circles indicate the individual value of each mouse and mean ± SEM is shown by bars. Significant differences are shown by (***) (*p* < 0.001).

A lack of IRAG1 caused a significant increase in spleen weights of the female IRAG1-KO mice compared to their WT littermates (Table 1, Figure 2A), and the same result was found in the total analysis of both sexes. Between male IRAG1-WT and their KO-littermates, no differences were found. The explored body weight of the genotypes and sexes differed insignificantly (Table 1, Figure 2B) and thus, did not influence the calculation of the spleen-weight-to-body-weight ratio. The spleen/body-ratio calculated from these weights was significantly increased in the female IRAG1-KO mice and in the total analysis of both sexes compared to the corresponding IRAG1-WT (Table 1, Figure 2C). No significant difference in this ratio was found between male IRAG1-WT and IRAG1-KO mice. In 7-week-old *Irag1*-deficient mice, no differences were detected in these parameters compared to their WT littermates (Figure S1B–D).

These results demonstrate that *Irag1*-deficiency led to an increase in the spleen weight and spleen-to-body-weight ratio. This phenotype was mainly observed in female IRAG1-KO mice.

### 2.3. Analysis of Hematological Parameters

To assess whether the gastrointestinal bleeding resulted in hematological abnormalities, we investigated in the following step the hematological status. Therefore, we characterized some basic hematological parameters, with the aim to obtain an overview of the hematological status of *Irag1*-deficient mice.

**Figure 3:**
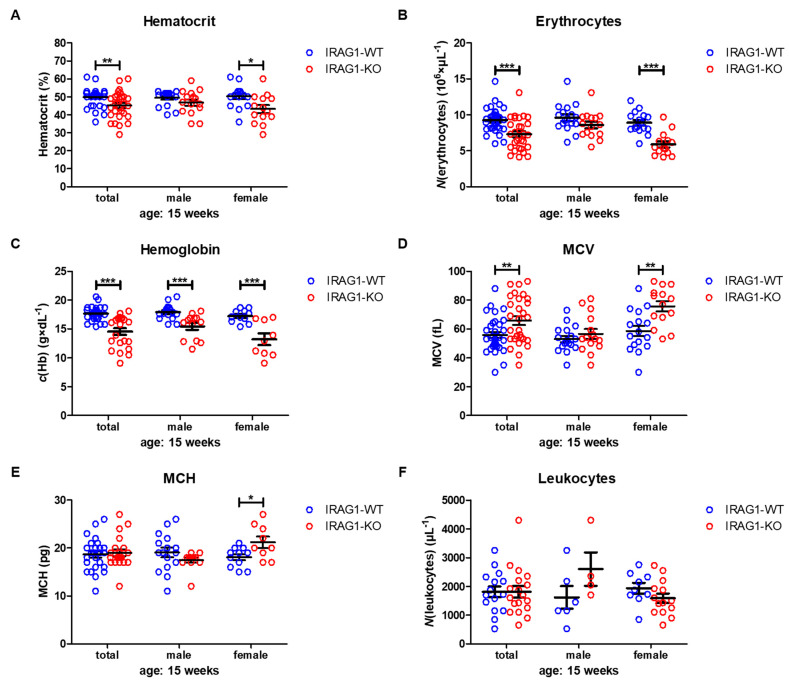
Hematological parameters of IRAG1-WT and IRAG1-KO mice. (**A**) Significant reduction of hematocrit in IRAG1-KO mice (total: *n* = 29; female: *n* = 14) in comparison to IRAG1-WT mice (total: *n* = 33 and female: *n* = 16) and no differences were detected in male IRAG1-WT (*n* = 17) and IRAG1-KO mice (*n* = 15). (**B**) *Irag1*-deficiency reduced RBCs in IRAG1-KO (total: *n* = 29; female: *n* = 14) and had no effect in male IRAG1-KO mice (*n* = 15) as compared with their WT littermates (total: *n* =33; male: *n* = 17; female: *n* = 16). (**C**) Significantly affected hemoglobin values of IRAG1-KO mice (total: *n* = 22; male: *n* = 13; female: *n* = 9) by the *Irag1*-deficiency compared to WT littermates (total: *n* = 27; male: *n* = 16; female: *n* = 11). (**D**) MCV was significantly increased in IRAG1-KO mice (female: *n* = 14; total: *n* = 29) compared to IRAG1-WT (female: *n* = 16; total: *n* = 33), and no difference was found between male IRAG1-WT (*n* = 17) and IRAG1-KO mice (*n* = 15). (**E**) MCH was not affected by *Irag1*-deficiency in male mice (IRAG1-WT: *n* = 16; IRAG1-KO: *n* = 13) and in total (IRAG1-WT: *n* = 27; IRAG1-KO: *n* = 22). Female IRAG1-KO mice (*n* = 9) developed a significantly higher MCH compared to their WT littermates (*n* = 11). (**F**) WBCs were not different between IRAG1-WT mice (total: *n* = 15; male: *n* = 6; female: *n* = 9) and IRAG1-KO mice (total: *n* = 18; male: *n* = 4; female: *n* = 14). Circles indicate the individual value of each mouse and mean ± SEM is shown by bars. Significant differences are shown by (*) (*p* < 0.05), (**) (*p* < 0.01) and (***) (*p* < 0.001).

Hematocrit (HCT) values were significantly reduced in female IRAG1-KO mice and in the total analysis of both sexes, while in male IRAG1-KO mice, no reduction was found (Table 2, Figure 3A). The number of erythrocytes/red blood cells (RBCs) was likewise significantly decreased in the total analysis and in female *Irag1*-deficient mice. Again, no significant difference was found in male IRAG1-KO compared to their WT littermates (Table 2, Figure 3B). A different result was found regarding the hemoglobin (Hb). In both sexes and in the total analysis of *Irag1*-deficient mice, significantly lower Hb levels were detected in contrast to IRAG1-WT mice (Table 2, Figure 3C). The mean corpuscular volume (MCV) (Table 2, Figure 3D) and mean corpuscular hemoglobin (MCH) (Table 2, Figure 3E) were elevated in female IRAG1-KO mice significantly and MCV in the total analysis in IRAG1-KO as well. Conversely, no differences in MCV and MCH were found in male mice between both genotypes. Finally, we analyzed the numbers of leukocytes/number of white blood cells (WBC), and we found no differences between both genotypes in both sexes (Table 2, Figure 3F). Seven-week-old IRAG1-WT and IRAG1-KO mice have no differences in these parameters (Figure S2). Old IRAG1-KO mice (age: 54–62 weeks) developed anemia and had an increase in the number of reticulocytes (Figure S3) and the reticulocytes production index (RPI) (Figure S4).

Taken together, the *Irag1*-deficiency caused severe anemia, as shown in the reduction of all important blood parameters. In this context, it was interesting to see that anemia was more prominent in female IRAG1-KO mice than in male IRAG1-KO mice.

### 2.4. IRAG1-KO Mice Exhibit an Iron Deficiency

In the next step, several parameters were researched to characterize the type of anemia in the IRAG1-KO mice.

**Figure 4:**
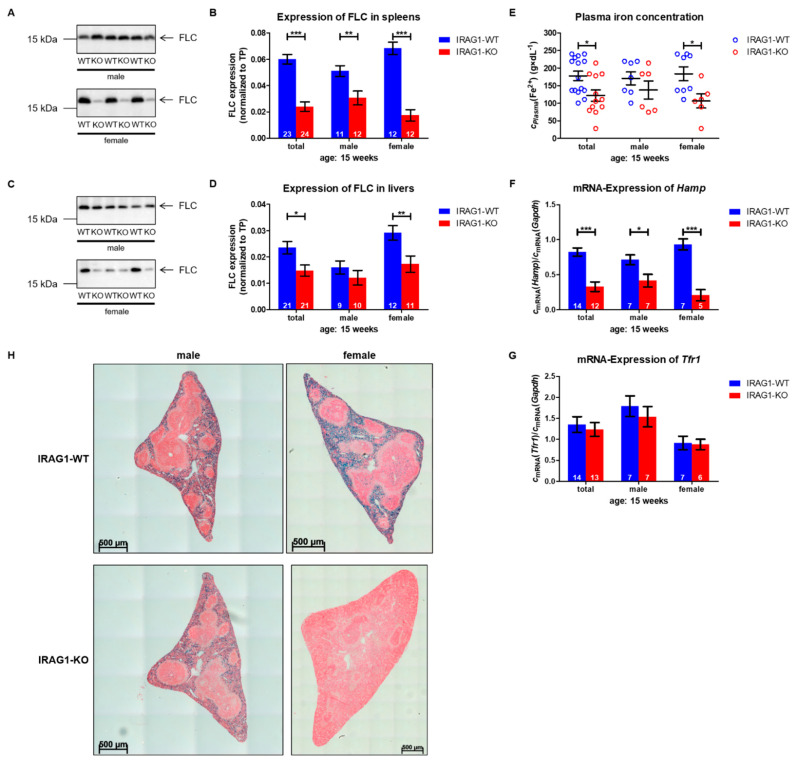
Iron deficiency in IRAG1-KO mice. (**A**,**B**) Representative ferritin light chain (FLC) expression (**A**) and statistical analysis of FLC expression (**B**) in the spleen of male and female IRAG1-WT (WT) and IRAG1-KO (KO) mice. (**C**,**D**) Representative FLC expression (**C**) and statistical analysis of FLC expression (**D**) in the liver of male and female IRAG1-WT and IRAG1-KO mice. (**E**) Reduced plasma iron (Fe^2+^) concentration in total and female IRAG1-KO mice (total: *n* = 12; female: *n* = 6) in relation to IRAG1-WT mice (total: *n* = 15; female: *n* = 8). Male IRAG1-KO (*n* = 6) exhibited no significantly lower plasma Fe^2+^ concentration compared to IRAG1-WT mice (*n* = 7). (**F**) Hepcidin-mRNA (*Hamp*) was significantly reduced in IRAG1-KO mice compared to the WT littermates. (**G**) mRNA expression of the transferrin receptor 1 (*Tfr1*) was not altered between IRAG1-WT mice and IRAG1-KO mice. (**H**) Iron staining (blue color) in spleens of representative sections of male and female IRAG1-KO and IRAG1-WT mice. Blue staining was detectable in male and female IRAG1-WT mice. Female IRAG1-KO mice had a strong reduction in detectable iron in the spleen, in contrast to male IRAG1-KO mice, which had only a slight reduction in the iron-staining compared to their WT littermates. The numbers in the graphs (**B**,**D**,**F**,**G**) indicate the mice that were investigated; the mean ± SEM is shown. The circles (**E**) indicate the individual value of each mouse, and the mean ± SEM is shown by bars. Significant differences are shown by (*) (*p* < 0.05), (**) (*p* < 0.01) and (***) (*p* < 0.001). Images of total protein (TP) were shown in Figure S5.

Western blot analysis of the expression of the ferritin light chain (FLC) in spleens was markedly decreased in IRAG1-KO mice of both sexes in comparison to IRAG1-WT mice (Figure 4A,B). Similar results were found in livers, in which the expression of FLC was significantly reduced in total and in female IRAG1-KO mice but not in the males (Figure 4C,D). The measurement of the concentrations of iron (Fe^2+^) in plasma exhibited a significant reduction of Fe^2+^ in female IRAG1-KO mice compared to WT littermates (IRAG1-WT: 183.91 µg × dL^−1^ ± 20.08 µg × dL^−1^ vs. IRAG1-KO: 106.56 µg × dL^−1^ ± 19.96 µg × dL^−1^) and in the analysis of both sexes (IRAG1-WT: 177.75 µg × dL^−1^ ± 13.37 µg × dL^−1^ vs. IRAG1-KO: 122.07 µg × dL^−1^ ± 16.15 µg × dL^−1^). In male IRAG1-KO mice, the reduction of the Fe^2+^ concentration (IRAG1-WT: 170.70 µg × dL^−1^ ± 18.43 µg × dL^−1^ vs. IRAG1-KO: 137.59 µg × dL^−1^ ± 25.56 µg × dL^−1^) was also measured, but this was not significant (Figure 4E). Further characterization of the anemia of the mRNA of livers of IRAG1-WT and IRAG1 mice were investigated regarding hepcidin (*Hamp*) and transferrin receptor 1 (*Tfr1*). *Hamp* expression was reduced in IRAG1-KO mice in the total as well as in both sexes in contrast to WT littermates (Figure 4F). In contrast to these findings, no alteration in the expression of *Tfr1* was detected between IRAG1-WT and IRAG1-KO mice in both sexes (Figure 4G). Finally, histological examinations of spleens of the IRAG1-WT and IRAG1-KO mice were performed with the Prussian blue staining to detect iron (Figure 4H). Iron (blue staining) was visible in the spleens of male and female IRAG1-WT mice. In *Irag1*-deficient mice, there were different results found. Spleens of female IRAG1-KO mice left no evidence of blue staining, instead of male mice, in which reduced staining of iron was visible.

These findings suggested that the anemia, which IRAG1-KO mice develop, leads to an iron deficiency that might be caused by the gastrointestinal bleeding.

### 2.5. Analysis of the Expression of Several cGMP/PKGI Signaling Proteins in IRAG1-WT and IRAG1-KO Mice

The similarity in the phenotypes between *Irag1* mutants and the global *Prkg1*-KO mice [9,10,11,12] led to the question if IRAG1-KO mice have a different protein expression of important proteins in the NO-cGMP-PKGI signaling pathway. Therefore colons, spleens and stomachs of IRAG1-KO and WT littermates were investigated via Western blotting.

**Figure 5:**
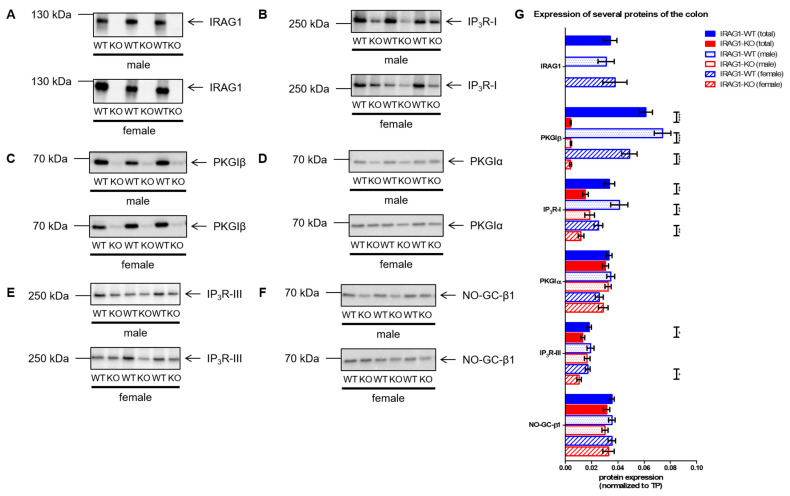
The expression of several cGMP/PKGI signaling proteins in colons from IRAG1-KO (KO) and IRAG1-WT (WT) mice. Protein expression of IRAG1-KO mice and their WT littermates (respective age: 15 weeks) were analyzed by western blot (**A**–**F**) and normalized to total protein (TP) for quantification (**G**). (**A**) Representative Western blot of IRAG1 in colons of male and female IRAG1-WT (total: *n* = 18; male: *n* = 10; female: *n* = 8) showed no differences between the sexes and no IRAG1 protein was found in IRAG1-KO mice (total: *n* = 22; male: *n* = 11; female: *n* = 11). (**B**) Western blot of IP_3_R-I showed a significant decrease in its expression in IRAG1-KO mice (total: *n* = 22; male: *n* = 11; female: *n* = 11) compared to WT littermates (total: *n* = 21; male: *n* = 11; female: *n* = 10). (**C**) The tremendous reduction of PKGIβ protein in IRAG1-KO mice (total: *n* = 22; male: *n* = 11; female: *n* = 11) compared to IRAG1-WT mice (total: *n* = 24; male: *n* = 12; female: *n* = 12). (**D**) PKGIα expression was not altered between IRAG1-KO (total: *n* = 20; male: *n* = 10; female: *n* = 10) and IRAG1-WT mice (total: *n* = 22; male: *n* = 11; female: *n* = 11). (**E**) Reduced IP_3_R-III expression in IRAG1-KO mice (total: *n* = 21; male: *n* = 10; female: *n* = 11) in relation to IRAG1-WT mice (total: *n* = 17; male: *n* = 9; female: *n* = 8). (**F**) No altered NO-GC-β1 expression between IRAG1-KO (total: *n* = 22; male: *n* = 11; female: *n* = 11) and IRAG1-WT mice (total: *n* = 23; male: *n* = 12; female: *n* = 11). (**G**) Statistical analysis of the expression of investigated proteins (**A**–**F**). The mean ± SEM is shown in the graphs, and significant differences are shown by (*) (*p* < 0.05), (**) (*p* < 0.01) and (***) (*p* < 0.001). Images of total protein (TP) were shown in Figure S6.

In colons of male and female IRAG1-WT mice, the IRAG1 protein was detected with no difference in protein expression between the sexes, and of course, no IRAG1 protein was found in IRAG1-KO mice (Figure 5A,G). The comparison of IP_3_R-I expression in IRAG1-KO mice and their WT littermates provided the finding of a reduced expression in IRAG1-KO mice overall sexes (Figure 5B,G). PKGIβ showed a tremendous and highly significant reduction (Figure 5C,G) in the samples of the IRAG1-KO mice regardless of gender, whereas PKGIα was not altered by the loss of IRAG1 (Figure 5D,G). Further, a slight reduction of IP_3_-receptor type III (IP_3_R-III) was found in IRAG1-KO mice, which was significant in the total analysis and female IRAG1-KO mice (Figure 5E,G). The expression of the nitric oxide (NO)-sensitive (soluble) guanylyl cyclase subunit β1 (NO-GC-β1) was not affected by the *Irag1*-deficiency (Figure 5F,G).

**Figure 6:**
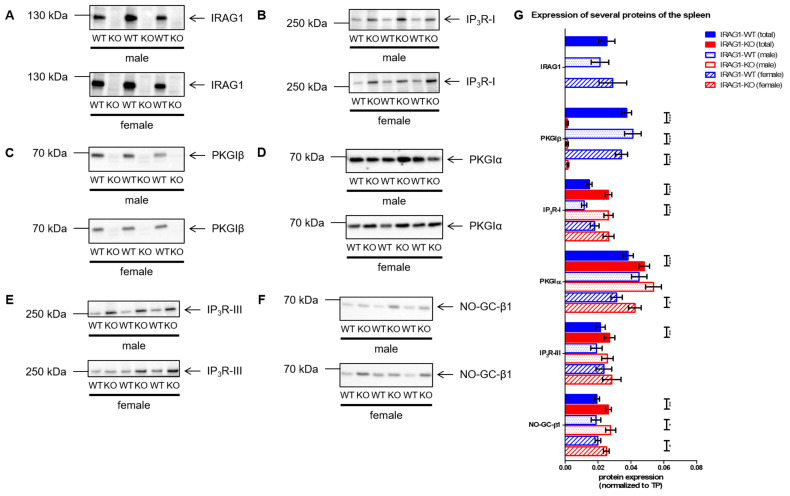
The expression of several cGMP/PKGI signaling proteins in spleens from IRAG1-KO (KO) and IRAG1-WT (WT) mice. Protein expression of IRAG1-KO mice and IRAG1-WT mice (respective age: 15 weeks) were analyzed by Western blot (**A**–**F**) and for quantification normalized to total protein (TP) (**G**). (**A**) Western blot of IRAG1 of male and female IRAG1-WT (total: *n* = 23; male: *n* = 11; female: *n* = 12) and IRAG1-KO mice (total: *n* = 24; male: *n* = 12; female: *n* = 12). (**B**) Increased expression of IP_3_R-I in IRAG1-KO mice (total: *n* = 22; male: *n* = 12; female: *n* = 10) compared to WT littermates (total: *n* = 22; male: *n* = 11; female: *n* = 11). (**C**) The expression of PKGIβ was reduced in IRAG1-KO mice (total: *n* = 22; male: *n* = 11; female: *n* = 11) compared with IRAG1-WT mice (total: *n* = 24; male: *n* = 12; female: *n* = 12). (**D**) PKGIα expression increased in IRAG1-KO mice (total: *n* = 24; male: *n* = 12; female: *n* = 12) in relation to their WT littermates (total: *n* = 22; male: *n* = 11; female: *n* = 11). (**E**): IRAG1-KO mice (total: *n* = 23; male: *n* = 12; female: *n* = 11) had an increase in IP_3_R-III expression than IRAG1-WT mice (total: *n* = 23; male: *n* = 11; female: *n* = 12). (**F**) Higher NO-GC-β1 expression was found in IRAG1-KO mice (total: *n* = 22; male: *n* = 11; female: *n* = 11) as in their WT littermates (total: *n* = 24; male: *n* = 11; female: *n* = 13). (**G**) Statistical analysis of the expression of investigated proteins (**A**–**F**). Mean ± SEM is shown in the graphs, and significant differences are shown by (*) (*p* < 0.05), (**) (*p* < 0.01) and (***) (*p* < 0.001). Images of total protein (TP) were shown in Figure S7.

Western blot analysis of spleens of IRAG1-WT and IRAG1-KO mice revealed similar alterations of the protein expression in colons. IRAG1 was not altered in the expression between male and female WT mice (Figure 6A,G). The expression of IP_3_R-I was upregulated in IRAG1-KO mice in contrast to their WT littermates, but only in the total analysis and in the male IRAG1-KO mice, this upregulation was significant (Figure 6B,G). As in colons, the expression of PKGIβ was drastically reduced in IRAG1-KO mice (Figure 6C,G), whereas PKGIα was expressed higher compared to IRAG1-WT mice (Figure 6D,G). As well as the IP_3_R-I, a higher expression of IP_3_R-III was detected, but in comparison to the IRAG1-WT mice, this was only significant in the common analysis (Figure 6E,G). For all sexes, NO-GC-β1 was more strongly expressed in IRAG1-KO mice than in WT mice (Figure 6F,G).

**Figure 7:**
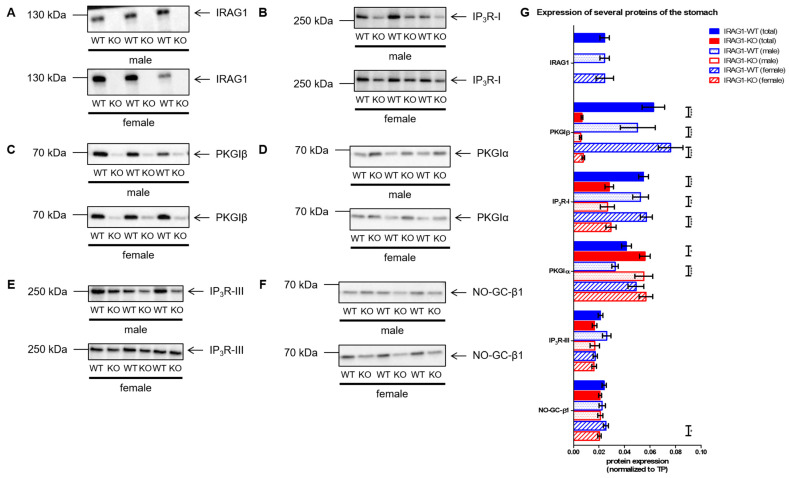
The expression of several cGMP/PKGI signaling proteins in stomachs from IRAG1-KO (KO) and IRAG1-WT (WT) mice. Protein expression of IRAG1-KO mice and their WT littermates (respective age: 15 weeks) were analyzed by Western blot (**A**–**F**) and normalized to total protein (TP) (**G**). (**A**) Western blot of IRAG1 in IRAG1-WT (total: *n* = 22; male: *n* = 12; female: *n* = 10) and IRAG1-KO mice (total: *n* = 23; male: *n* = 11; female: *n* = 12). (**B**) Decreased expression of IP_3_R-I in IRAG1-KO mice (total: *n* = 22; male: *n* = 12; female: *n* = 10) compared to IRAG1-WT mice (total: *n* = 22; male: *n* = 12; female: *n* = 10). (**C**) The expression of PKGIβ was reduced in male and female IRAG1-KO mice (total: *n* = 23; male: *n* = 11; female: *n* = 12) in relation to WT littermates (total: *n* = 23; male: *n* = 12; female: *n* = 11). (**D**) Increased PKGIα expression was detected in IRAG1-KO mice (total: *n* = 21; male: *n* = 10; female: *n* = 11) towards their WT littermates (total: *n* = 24; male: *n* = 11; female: *n* = 13). (**E**) No difference in expression of IP_3_R-III between IRAG1-WT mice (total: *n* = 24; male: *n* = 11; female: *n* = 13) and IRAG1-KO mice (total: *n* = 22; male: *n* = 10; female: *n* = 12) were found. (**F**) Stomachs of IRAG1-KO mice (total: *n* = 23; male: *n* = 11; female: n = 12) indicated a decrease in NO-GC-β1 expression in relation to WT littermates (total: *n* = 25; male: *n* = 12; female: *n* = 13). (**G**) Statistical analysis of the expression of investigated proteins (**A**–**F**). The mean ± SEM is shown in the graphs, and significant differences are shown by (*) (*p* < 0.05), (**) (*p* < 0.01) and (***) (*p* < 0.001). Images of total protein (TP) were shown in Figure S8.

The analysis of cGMP/PKGI signaling proteins in the stomach showed similar results, too. Similar to the colon and the spleen, no differences in the expression of IRAG1 in male and female IRAG1-WT mice were found (Figure 7A,G). The expression of the IP_3_R-I was significantly reduced in both sexes in IRAG1-KO mice compared to their WT littermates (Figure 7B,G). Similar to in colons and spleens of IRAG1-KO mice, a strongly reduced expression of PKGIβ was detected (Figure 7C,G). Indeed, an upregulation of the PKGIα was found in the IRAG1-KO mice, which was significantly different in the total and male IRAG1-KO mice in relation to IRAG1-WT mice (Figure 7D,G). The expressions of the IP_3_R-III (Figure 7E,G) and NO-GC-β1 (Figure 7F,G) were not influenced by the loss of IRAG1.

Taken together, *Irag1*-deficiency caused a massive reduction of the PKGIβ expression and changes the expression of several cGMP/PKGI signaling proteins depending on the tissue.

### 2.6. Analysis of mRNA Levels of cGMP/PKGI Signaling Genes in Colon, Spleen and Stomach

The results of the analysis of the altered protein expression of several cGMP/PKGI signaling proteins in the investigated tissues led to questioning if alterations in the protein expression are gene-related effects, and therefore, mRNA was analyzed.

**Figure 8:**
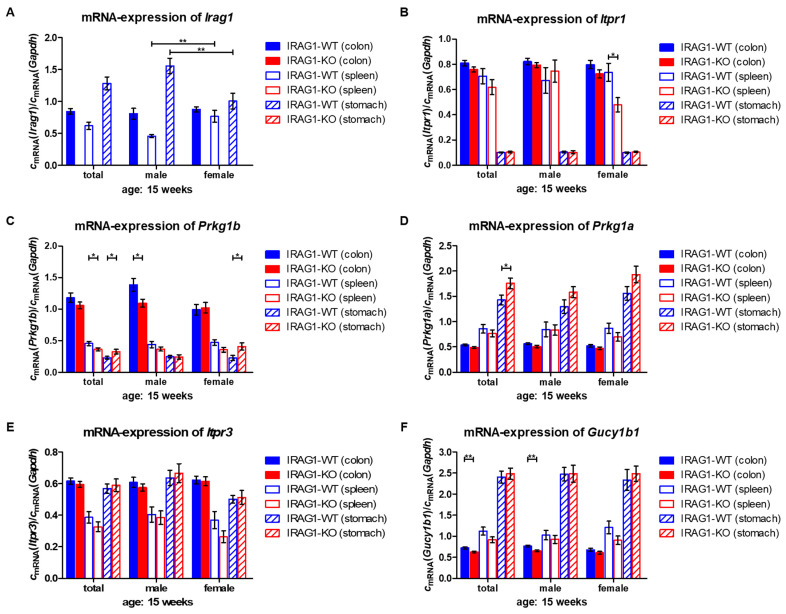
mRNA expression of several cGMP/PKGI-signaling genes from IRAG1-WT and IRAG1-KO mice. mRNA levels of relevant cGMP/PKGI-signaling genes were analyzed in colon, spleen and stomach of IRAG1-WT and IRAG1-KO mice (respective age: 15 weeks). (**A**) *Irag1* mRNA was not found in IRAG1-KO mice (colon: total: *n* = 24, male: *n* = 12, female: *n* = 12; spleen: total: *n* = 23, male: *n* = 12, female: *n* = 11; stomach: total: *n* = 24, male: *n* = 12, female: *n* = 12). Differed mRNA expression of *Irag1* in spleens and stomachs between male and female of IRAG1-WT mice (colon: total: *n* = 23, male: *n* = 11, female: *n* = 12; spleen: total: *n* = 23, male: *n* = 11, female: *n* = 12; stomach: total: *n* = 24, male: *n* = 12, female: *n* = 12). (**B**) No differences were found in mRNA levels of *Itpr1* in colon and stomach (IRAG1-WT: colon: total: *n* = 23, male: *n* = 11, female: *n* = 12; stomach: total: *n* = 24, male: *n* = 12, female: *n* = 12; IRAG1-KO: total: *n* = 24; male: *n* = 12; female: *n* = 12; stomach: total: *n* = 24, male: *n* = 12, female: *n* = 12). Spleens of IRAG1-KO mice (total: *n* = 23, male: *n* = 12, female: *n* = 11) showed a reduction compared to the WT littermates (total: *n* = 23, male: *n* = 11, female: *n* = 12). (**C**) Diminished expression of *Prkg1b* mRNA in colon and spleen of IRAG1-KO mice (colon: total: *n* = 24, male: *n* = 12, female: *n* = 12; spleen: total: *n* = 23, male: *n* = 12, female: *n* = 11) in relation to IRAG1-WT mice (colon: total: *n* = 23, male: *n* = 11, female: *n* = 12; spleen: total: *n* = 23, male: *n* = 11, female: *n* = 12). In stomachs of IRAG1-KO mice (total: *n* = 24, male: *n* = 12, female: *n* = 12), *Prkg1b* mRNA was slightly increased compared to IRAG1-WT mice (total: *n* = 24, male: *n* = 12, female: *n* = 12). (**D**) mRNA of *Prkg1a* was not influenced by the loss of IRAG1 in colon (IRAG1-WT: total: *n* = 23, male: *n* = 11, female: *n* = 12; IRAG1-KO: total: *n* = 23, male: *n* = 12, female: *n* = 11) and spleen (IRAG1-WT: total: *n* = 23, male: *n* = 11, female: *n* = 12; IRAG1-KO: total: *n* = 23, male: *n* = 12, female: *n* = 11). In stomachs of IRAG1-KO mice, a slight increase was detected (IRAG1-WT: total: *n* = 24, male: *n* = 12, female: *n* = 12; IRAG1-KO: total: *n* = 22, male: *n* = 12, female: *n* = 12). (**E**) Expression of *Itpr3* mRNA was changed by the loss of IRAG1 in the investigated tissues (IRAG1-WT: colon: total: *n* = 23, male: *n* = 11, female: *n* = 12; spleen: total: *n* = 23, male: *n* = 11, female: *n* = 12; stomach: total: *n* = 24, male: *n* = 12, female: *n* = 12; IRAG1-KO: total: *n* = 24, male: *n* = 12, female: *n* = 12; spleen: total: *n* = 23, male: *n* = 12, female: *n* = 11; stomach: total: *n* = 24, male: *n* = 12, female: *n* = 12). (**F**) mRNA levels of *Gucy1b1* were not different between IRAG1-WT mice (IRAG1-WT: colon: total: *n* = 23, male: *n* = 11, female: *n* = 12; spleen: total: *n* = 23, male: *n* = 11, female: *n* = 12; stomach: total: *n* = 24, male: *n* = 12, female: *n* = 12) and IRAG1-KO mice (total: *n* = 24, male: *n* = 12, female: *n* = 12; spleen: total: *n* = 23, male: *n* = 12, female: *n* = 11; stomach: total: *n* = 24, male: *n* = 12, female: *n* = 12). The mean ± SEM is shown in the graphs, and significant differences are shown by (*) (*p* < 0.05), (**) (*p* < 0.01).

The mRNA levels of IRAG1 (*Irag1*) in IRAG1-WT showed significant differences between male and female mice in the spleen and stomach but not in the colon (Figure 8A). IP_3_R-I mRNA (*Itpr1*) was not affected by the loss of IRAG1 in the colon and stomach, but a significant reduction in female spleens of IRAG1-KO mice was found (Figure 8B). Colonic mRNA of *Prkg1b* was not altered in total and in female IRAG1-KO mice but slightly and significantly diminished in male IRAG1-KO mice (Figure 8C). Analysis of the spleen showed a similar result to the colon, but in this tissue, only a tiny reduction of *Prkg1b* mRNA was revealed in the common analysis IRAG1-KO mice. In contrast, a slight increase in the mRNA levels of the stomach was found in total and female IRAG1-KO mice, but no differences were found in male mice. Colonic and splenic mRNA levels of *Prkg1a* were not altered between IRAG1-WT and IRAG1-KO mice. However, an upregulation was identified in the stomach of IRAG1-KO mice, which was only significant in the total analysis (Figure 8D). mRNA levels of the IP_3_R-III (*Itpr3*) were not altered between IRAG1-WT and IRAG1-KO in all investigated tissues (Figure 8E). Similar results were observed for *Gucy1b1* (Figure 8F). No differences were recognized between the genotypes in the spleen and the stomach. Only in the colon, a small but significant reduction of the mRNA was detected in the total analysis and in male IRAG1-KO mice.

These results show that *Irag1*-deficiency exhibits no effects on the mRNA expression of several genes of the NO-cGMP-signaling pathway.

Taken together, *Irag1*-deficiency indicates that gastrointestinal bleedings lead to an iron deficient anemia, which is accompanied by splenomegaly. However, these results are more dominant in female than in male mice. Furthermore, lead the *Irag1*-deficiency to a reduction of PKGIβ protein. 

## 3. Discussion

IRAG1 is a protein, which is expressed in a lot of smooth muscle-containing tissues [5], and a loss or defect of IRAG1 causes gastrointestinal abnormalities [4,8]. Analysis of the feces of IRAG1-KO mice exhibited gastrointestinal bleeding and anemia, which were more dominant in female mice. Global *Prgk1* mutants have similar gastrointestinal phenotypes [9] and develop gastrointestinal bleeding, which can be cured or prevented with the treatment of PPI [11,12], indicating that the gastrointestinal bleeding is caused by duodenal ulcerations that were induced by a dysfunction of pH regulation in the duodenum. It has been proven that IRAG1 has an important role in the pathophysiological function of the gastrointestinal tract. MRVI1—the human homologue of IRAG1—exhibits a role in the development of achalasia, which can cause injuries of the mucous membrane [13,14]. Therefore, it is possible that a loss of IRAG1 results in gastrointestinal lesions or microbleeding because no duodenal ulcerations—as described in global *Prkg1* mutants—were detected in *Irag1*-deficient mice (Figure S9). Another reason for the bleeding could be a kind of obstipation as a consequence of the retarded gastrointestinal passage time of IRAG1-KO mice [4], which led to dry and harder feces. This might provoke local injuries in the bowel, which are the cause of the bleeding. The Haemoccult^®^ Test is only a method to identify fecal occult blood. In concrete terms, the test detects blood from the beginning of the gastrointestinal tract (esophagus) to the end (rectum), and it is not possible to localize the source of the bleeding with this method. Admittedly not only mice that lack IRAG1 or PKGI develop such gastrointestinal abnormalities. Mice lacking the β_1_ subunit of NO-GC (GC-KO) suffered similar gastrointestinal symptoms and have a reduced life span [15], whereas a loss of α_1_ or α_2_ subunit did not lead to such symptoms [16]. Reticulocytes and RPI were increased in IRAG1-KO mice and might be the cause for the increased MCV and MCH levels in those mice. However, these results demonstrate that *Irag1*-deficiency led to an adequate regeneration of the blood. This is in line with the measured folate concentration of the plasma concentration of IRAG1-KO mice, which also were not reduced (Figure S10). Therefore, the described anemia is not caused by inadequate erythropoiesis. The comparison between the blood parameters of IRAG1-KO mice and global *Prkg1*-KO mice [10,12] shows that IRAG1-KO mice suffer from a milder form of anemia. Furthermore, global *Prkg1*-KO mice have a strongly reduced survival time and age-dependent systemic hypertension [9], in contrast to IRAG1-KO mice [8,17]. In this context, it is important to mention that mice with a selective mutation in the N-terminal protein interaction domain of PKGIα (LZM mice) do not develop anemia and have no reduced life span [18], contrary to global *Prkg1*-KO mice. These results lead to the presumption that PKGIβ/IRAG1 signaling may play an important role in the development of these phenotypes. Taken together, IRAG1-KO mice suffer from anemia, which is caused by (chronic) gastrointestinal bleeding.

Often iron deficiency is caused by anemia as a consequence of gastrointestinal bleeding. Reduced levels of hemoglobin are the first hint that there is a suspicion of anemia and iron deficiency, and it is a good marker for the follow up [19,20,21]. All our results bring us to the conclusion that IRAG1-KO mice have an iron deficiency—reduced FLC levels in the liver and spleen, reduced plasma iron concentration, which indicates that iron storages are empty. The strong reduction of FLC expression is an indicator for empty iron storages, which is supported by the reduced plasma concentration of iron, and it is visible in the histological examinations of spleens via the Prussian blue staining. Another indicator of an iron deficiency is the strong reduction of mRNA of the hepcidin gene in *Irag1*-deficient mice. The function of hepcidin is to regulate the iron uptake/liberation into the body. Low levels of hepcidin indicate that the body has a deficit in iron [22]. Our findings are in accordance with the findings in global *Prkg1*-KO mice, which developed an iron deficiency caused by anemia as a follow of the gastrointestinal bleedings [12], which why we assume (chronic) blood loss as the direct cause of the iron deficiency in global *Irag1*-deficient mice.

Causes of splenomegaly are multiple—one of them is anemia. *Irag1*-deficiency led to gastrointestinal bleeding and anemia but also to splenomegaly. In the first description of splenomegaly and anemia in global *Prkg1*-KO mice, a reduced life span of erythrocytes was mentioned [10] and justified with the expression of the PKGI in erythrocytes. In the literature, there is a controversy about the PKGI expression in erythrocytes. Föller et al. and Cortese-Krott et al. postulated the expression of the PKGI in erythrocytes [10,23], and Angermeier et al. found no expression of the PKGI in erythrocytes [12]. This is important for the question of the functional role of IRAG1 in this context. IRAG1 expression was analyzed in erythrocytes as part of the expression analysis of PKGI in these, and no IRAG1 could be detected [12]. It is a scientific consensus that erythrocytes lose their cell organelles in the process of maturation [24,25,26]. So, erythrocytes will lose amongst other cell organelles their endoplasmic reticulum. In the first description of MRVI1, this protein was not detected in erythroleukemia cell line K562, which can spontaneously develop characteristics to early-stage erythrocytes [27]. This leads to the assumption that it is very unlikely that a malfunction of erythrocytes caused by the loss of IRAG1 is the reason for the splenomegaly. On the contrary, it seems very possible that the splenomegaly in IRAG1-KO mice is the result of (iron deficiency) anemia. In *Prkg1*-deficient mice, anemia and splenomegaly were reported [10,12], and with the progression of the anemia, a progression of the splenomegaly was found [10]. Treatment with PPI normalized the blood parameters so that global *Prkg1* mice show no iron deficiency anemia and splenomegaly [12]. This finding supports the hypothesis that splenomegaly is caused by gastrointestinal bleeding and not by the malfunctions of the erythrocytes. With chronic blood loss, the body developed a negative iron balance, which led to iron deficiency anemia. This phenomenon was observed in the present work upon the loss of IRAG1 and previously in global *Prkg1*-deficient mice [12]. The clinical evidence of anemia and splenomegaly caused by an iron deficiency is shown in mice, in which a targeted deletion of intestine divalent metal-ion transporter-1 (DMT1) has taken place [28]. Those DMT1^int/int^ mice developed all the classic symptoms of anemia, including reduced hepcidin levels and developed splenomegaly. An induced iron deficiency, e.g., by feeding low-iron-containing chow, results also in anemia. The mechanism of this finding is an enhanced programmed cell death of erythrocytes or eryptosis, e.g., by an increased expression of phosphatidylserine, which mediates the binging of macrophages [29]. Some similar results were found in the erythrocytes of *Prkg1*-deficient mice [10]. However, it is not expected that this mechanism is mediated by *Irag1*-deficiency. Hence, loss of IRAG1 causes gastrointestinal bleeding and, as a consequence, regenerative anemia, which is followed by an iron deficit caused by chronic blood loss. We suggest that iron deficiency leads to splenomegaly, which is therefore probably a secondary effect of the anemia. Another possible reason for splenomegaly is a cardiac malfunction caused by pulmonary arterial hypertension (PAH). It was described that patients with an idiopathic and heritable PAH could develop splenomegaly [30]. These reports are interesting because recent results about IRAG1 showed that IRAG1-KO mice developed pulmonary hypertension [17]. To what extent these findings are relevant in the development of the splenomegaly in IRAG1-KO mice has to be investigated in a further step, e.g., in combination with the feeding of PPI and/or iron supplementation.

Remarkably, *Irag1*-deficiency causes a similar phenotype to global *Prkg1* mice. IRAG1 forms a complex with PKGIβ and IP_3_R-I [1], is phosphorylated by PKGIβ and has a similar expression pattern to this enzyme [5]. However, we did not expect this similarity in the results. Hence, we investigated the expression of several cGMP/PKGI-signaling proteins in IRAG1-KO mice. The reduction of the protein levels of PKGIβ in *Irag1*-deficient mice was tremendous and not expected. In previous results, a reduction of PKGIβ was found in the aorta and colons of *Irag1^Δ12/Δ12^* mice [4] and global IRAG1-KO mice (but not quantified) [8].Therefore, our findings are also in line with former data and recently published results of the PKGIβ expression in the lung and right ventricle of the heart [17]. To find out what could be the reason for these reduced PKGIβ protein levels, we analyzed the PKGIβ mRNA levels, and this data showed no reduction of mRNA levels in IRAG1-KO mice. Hence, reduced protein levels of PKGIβ are not caused by a reduced gene expression. However, PKGIβ protein levels in *Irag1*-deficient mice are reduced in all tissues, in which IRAG1 would be expressed. In contrast to global *Irag1*-deficient mice, the PKGIβ protein was not reduced in LZM mice [18,31]. Derived from these findings, it is suggested that IRAG1 is important for the stability of PKGIβ protein by forming its interaction complex. Therefore, a loss of IRAG1 led to instability of PKGIβ protein. From the PKGIα protein, it is known that intense ubiquitination is a mechanism of their degradation [32]. This could be also a possible mechanism of degradation of PKGIβ protein. The expression of PKGIα, which is no interaction partner of IRAG1, was not altered in colons of IRAG1-KO mice, but we found an increase in spleens and stomachs and higher mRNA levels were only found in stomachs of IRAG1-KO mice. In the analysis of lungs and the right ventricle of IRAG1-KO mice, there was no alteration of PKGIα protein found [17]. So, our results are in line with previous results. The presence of PKGIα has no positive effect on the pathological function of an *Irag1*-deficiency because it is not able to compensate for the loss of PKGIβ function. On the contrary, lack of PKGIα in LZM mice has no negative impact on their life span or their blood parameters [18]. With these results and the similar phenotypes of IRAG1-KO mice to global *Prkg1*-deficient mice [9,10,11,12], we can hypothesize that, in global *Prkg1*-deficient mice, the loss of PKGIβ is a decisive factor for the described phenotypes in these mice.

IP_3_R-I protein expression was reduced in colons and stomachs of IRAG1-KO mice. The reduced levels are confirmed by expression analysis of *Irag1^Δ12/Δ12^* mice, in which a slight reduction of IP_3_R-I was found [4] and in the first expression analysis of the global IRAG1-KO mice [8]. This data suggests that the reduction of IP_3_R-I is not caused by transcriptional effects in accordance with the data from PKGIβ. It is a further hint that IRAG1 is important for the stability of its interaction proteins, namely IP_3_R-I and PKGIβ. The increase in IP_3_R-I in IRAG1-KO spleens is analogues to the findings in the lung and the right ventricle of hearts of IRGA1-KO mice, where higher protein levels of IP_3_R-I compared to WT mice were described [17]. IP_3_R-III and NO-GC-β1 were not influenced by the loss of IRAG1. 

Changes in the expression of cGMP-signaling components were similar in male and female IRAG1-KO mice, but phenotype changes of female IRAG1-KO mice were stronger than of male IRAG1-KO mice. Although the reason for this gender difference is not clear, it is interesting that cGMP-related gender effects, e.g., estrogen in the gastrointestinal tract, were already observed in other studies [33]. Furthermore, iron deficiency and anemia in women are global health problems [34]. Hence, there might be gender-specific differences in the regulation of the gastrointestinal tract and/or iron-uptake/loss.

Recently, it was reported that sGC stimulators are promising substances for the treatment of sickle cell anemia [35]. Hence, cGMP signaling is relevant for different forms of anemia. However, cGMP modulators such as sGC stimulators and PDE5 inhibitors can also induce bleeding by inhibition of platelet function [36,37]. This must be considered in the therapy. These cGMP modulating substances could be also tested in further investigations regarding anemia and splenomegaly in IRAG1-KO mice.

**Figure 9:**
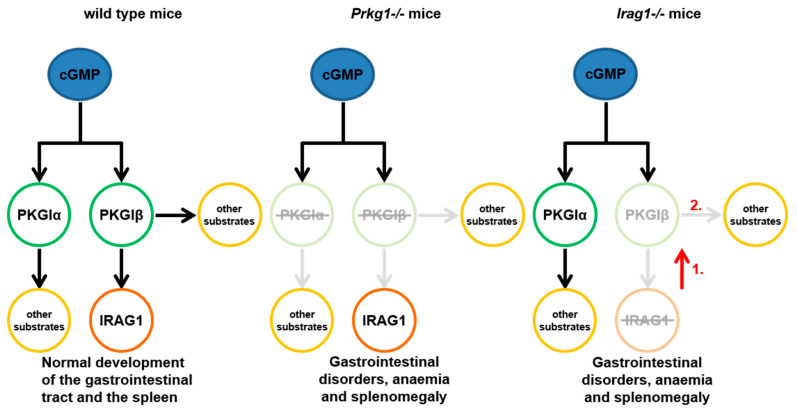
The effect of PKGIβ/IRAG1 signaling on pathophysiological functions. Wild-type mice have a normal expression of the PKGIα, PKGIβ and IRAG1. Thus, these mice have a normal gastrointestinal function and develop no anemia and splenomegaly. Global *Prkg1*-deficient mice (*Prkg1^−/−^*) developed all these symptoms [9,12] presumed by the loss of stimulation of their substrate proteins, e.g., IRAG1. *Irag1*-deficient mice (*Irag1^−/−^*) developed gastrointestinal disorders, anemia and splenomegaly, as well as *Prkg1*-deficient mice. Indeed, the loss of IRAG1 causes a reduction of PKGIβ protein (1) with the consequence of a reduced stimulation of other substrate proteins of the PKGIβ (2), while protein expression of PKGIα is not altered to wild type mice. Crossed out enzymes/proteins indicate genetically deleted enzymes/proteins, and light colors symbolize inactive or deficient signaling pathways.

Taken together with this present work, we demonstrate new results about the pathophysiological functions of IRAG1 (Figure 9):

1. A loss of IRAG1 causes gastrointestinal bleedings, anemia, iron deficiency and splenomegaly, with a dominant phenotype in female IRAG1-KO mice. Unfortunately, it is at the moment not possible to explain the difference between the sexes.
2. With this present work, we can demonstrate that IRAG1 is of prime importance for the stability of the PKGIβ protein and hence for PKGIβ/IRAG1 signaling.
3. Presence of PKGIα in IRAG1-KO mice cannot compensate for the functional loss of PKGIβ. These findings suggest the hypothesis that loss of PKGIβ/IRAG1 signaling is mainly responsible for the development of gastrointestinal bleedings, anemia and splenomegaly in global *Prkg1*-deficient mice.
4. IRAG1-KO mice are a kind of PKGIβ protein-deficient mouse; therefore, a possible model to study the pathophysiological function of PKGIβ in the presence of PKGIα.

## 4. Materials and Methods

### 4.1. Animal

Global *Irag1*-deficient mice were generated as described before [8]. For all following experiments, 14-week-old IRAG1-KO (genotype: *Irag1^−/−^*) mice and their WT littermates (IRAG1-WT; genotype: *Irag1^+/+^*) were used. IRAG1-KO mice have no reduced lifetime [17] and normal development. No differences were seen in the distribution of sexes (Table S1) and the Mendel ratio of the genotypes (Table S2).

The animals were bred and kept under specific-pathogen-free (SPF) conditions according to the guidelines of the Federation of European Laboratory Animal Science Associations in the animal facility of Regensburg University (Bavaria, Germany; registration: Regierung von Unterfranken: RUF-55-2.2-2535-2-1205-18) with free access to food and water ad libitum, following to all guidelines according to the German animal protection law.

### 4.2. Detection of Occult Blood

Male and female IRAG1-KO and -WT mice underwent the Haemoccult^®^ Test (Beckman Coulter GmbH, PZN: 1933716, Krefeld, Germany) at the age of 14 weeks. For the test, feces from each mouse was collected in accordance with the manufacturer’s instructions. This procedure was repeated in the following 2 days. The Haemoccult^®^ Test was considered positive if there was blue staining visible. Over the period of testing, the genotype of the mice was not known by the investigator.

### 4.3. Analysis of Haematological Parameters

For measurement of the blood parameters, mice were CO_2_-euthanized, and blood was drawn from the retrobulbar plexus. Hematocrit was determined by taking blood with hematocrit capillary (BRAND GMBH + CO KG, Wertheim, Germany) at the retrobulbar plexus and then centrifuged with 11,500× *g* for eight minutes at room temperature (RT) [38].

The number of erythrocytes (RBC) and leukocytes (WBC) were determined by filling specific pipettes with blood, and 0.9% NaCl solution (RBC) or “Türk’s solution” (Merck KGaA, Darmstadt, Germany) (WBC) was added. Cells were counted by using a “Neubauer improved” counting chamber, and a 40× objective (RBC) or a 10× objective (WBC) of the microscope was used. Finally, the concentration of RBC or WBC was calculated according to the literature [38].

Hemoglobin capillary (BRAND GMBH + Co KG, Wertheim, Germany) was filled with 20 µL of blood, added to 5 mL of the “transformation solution” (0.02% K_3_Fe(CN)_6_, 0.005% KCN, 0.014% KH_2_PO_4_ in ddH_2_O) and incubated for 15 min at RT. The extinction of the solution was measured against the “transformation solution” (λ = 546 nm), and the concentration of hemoglobin was calculated [38].

MCH and MCV were calculated with the blood parameters measured before [38].

### 4.4. Measurement of Plasma Iron Concentration

Blood was collected in a heparinized Microvette^®^ (SARSTEDT & Co, Nümbrecht, Germany) after centrifugation with 2000× *g* for 8 min at RT, and the isolated plasma was stored at −80 °C. The determination of the plasma iron concentration was conducted with the QuantiChrome^TM^ Iron Assay Kit (BIOTREND Chemikalien GmbH, Cologne, Germany) in accordance with the instructor’s manual. 

### 4.5. Removal of Spleen and Determination of Different Weights

Before the blood was collected, the body weight of the mice was determined. After blood collection, the removal of the spleen and spleen weight determination occurred. Afterwards, spleens were stored in accordance with the following procedures.

### 4.6. Tissue Preparation for Western Blot

Tissue samples, which were snap-frozen in liquid nitrogen and then stored at −80 °C, were homogenized with lysis buffer (2% nonaethylene glycol monododecyl ether, 150 mM NaCl, 20 mM Tris in ddH_2_O, pH = 8.0) containing proteinase inhibitors (1 mM benzamidine, 0.5 µg ×∙mL^−1^ leupeptin, 300 µM PMSF) and 1× PhosSTOP (Roche Diagnostics GmbH, Mannheim, Germany). The homogenate was centrifuged, and the supernatant was collected. The protein concentration was determined by a Lowry kit (Bio-Rad Laboratories, Inc., Munich, Germany), and the samples were then stored at −80 °C.

### 4.7. Western Blot Analysis

The expression of several proteins was analyzed by Western blotting in separate runs for each tissue. 50 µg of protein were used and blotted on a PVDF-membrane (Immobilon-P, Merck KGaA, Darmstadt, Germany) after electrophoresis. The membrane was incubated in the primary antibody (anti-PKGIα (in-house production): 1:80 [5]; anti-PKGIβ (in-house production): 1:200 [5]; anti-FLC (abcam): 1:2000; anti-IP_3_R-I (Cell signaling): 1:250, anti-IP_3_R-III (BD Transduction Laboratories^TM^, BD Biosciences, San Jose, USA): 1:100; anti-IRAG1 (in-house production): 1:200 [2]; anti-sGC-β-1 (NO-GC-β1) (Sigma-Aldrich^®^, Taufkirchen, Germany): 1:500) overnight. Detection was carried out with Clarity^TM^ Western ECL Substrate (Bio-Rad Laboratories, Inc., Munich, Germany) and ChemiDoc^TM^ MP Imaging System (Bio-Rad Laboratories, Inc., Munich, Germany) by using the imaging software Image Lab^TM^ Software (Bio-Rad Laboratories, Inc., Munich, Germany), after incubation in HRP-conjugated secondary antibody (anti-mouse (Sigma-Aldrich^®^, Taufkirchen, Germany): 1:10,000; anti-rabbit (Dianova GmbH, Hamburg, Germany): 1:10,000). For statistical analysis, the signal intensity has been normalized to the total protein of each lane.

### 4.8. Histology

Pieces of spleens were incubated overnight in 3% PFA-solution (3% PFA, 1 mM EGTA, 15 mM K_2_HPO_4_∙3H_2_O, 2 mM MgCl_2_∙6H_2_O, 90 mM NaCl, 100 mM sucrose in ddH_2_O, pH = 7.4) by 4 °C, removed on the next day and stored in 70% MeOH by 4 °C. Dehydration and embedding of the spleens were performed based on previous protocols [39], 4 µm serial sections of spleens were performed with a microtome (Thermo Scientific, Braunschweig, Germany) by using polylysine slides (Thermo Scientific, Braunschweig, Germany). The iron-staining slides have to be rehydrated (2 × 10 min xylene, 3 × 5 min isopropanol, 2 × 5 min 100% methanol, 2 min ddH_2_O). In the next step, the slides were incubated for 5 min in aqueous 10% K_4_[Fe(CN)_6_], followed by 30 min 5% K_4_[Fe(CN)_6_] in 3,7% HCl. Slides were washed (3 × 2 min) in ddH_2_O and counter-stained in 0.6% safranine O in 66% EtOH for 15 s and again washed (2 × 2 min) in ddH_2_O. Differentiation of the colors and dehydration of the slides was conducted by incubation in 80% EtOH for 2 min and 3 min 100% EtOH, followed by a final step of 5 min xylene and sections were embedded with DePeX (Serva GmbH, Heidelberg, Germany). Pictures of the slides were taken with Axiovert 200 microscope (Carl Zeiss AG, Jena, Germany) using the MosaiX module (Carl Zeiss AG, Jena, Germany) and analyzed with AxioVision Software (Carl Zeiss AG, Jena, Germany).

### 4.9. Isolation of RNA and qRT-PCR of Murine Tissues

Tissue samples were stored in RNA*later*^TM^ (Sigma-Aldrich^®^, Taufkirchen, Germany) overnight at 4 °C and then at −80 °C. RNA isolation was carried out with the PeqGOLD TriFast^TM^ reagent (PeqLab, Erlangen, Germany), and cDNA-synthesis was performed with 2 µg RNA according to in-house standard protocol [40]. For qRT-PCR, 2 µL of cDNA and the LightCycler^®^ 480 system (Roche Molecular Systems, Inc., Mannheim, Germany) was used as fluorescent dye SYBR green I (Roche Molecular Systems, Inc., Mannheim, Germany). In separate runs for each tissue, the mRNA expression of *Gucy1b1*, *Hamp*, *Irag1*, *Itpr1*, *Itpr3*, *Prkg1a*, *Prkg1b* and *Tfr1* was analyzed. *Gapdh* was used as a housekeeper. The sequences of specific primers are listed in Table S3. The evaluation was carried out by absolute quantification. Therefore, a standard curve of a dilution series was created from a known sample, and the ratio between the concentration of the gene and the concentration of *Gapdh* was calculated [40].

### 4.10. Statistical Analysis

All data are shown with mean ± SEM. For the statistical analysis, the Student’s *t*-test was used and calculated with “Graphpad Prism” and “Microsoft Excel.” Significant differences in the graphs are shown by (*) (*p* < 0.05), (**) (*p* < 0.01) and (***) (*p* < 0.001).

## Figures and Tables

**Table 1:** Spleen weight, body weight and spleen-weight-to-body-weight ratio (spleen/body-ratio) of total (male and female), male and female IRAG1-WT and IRAG1-KO mice.

Parameter	Total		Male		Female	
	*IRAG1-WT*	*IRAG1-KO*	*IRAG1-WT*	*IRAG1-KO*	*IRAG1-WT*	*IRAG1-KO*
spleen weight (g)	0.0634 ± 0.0034	0.1051 ± 0.0106 ^a^	0.0605 ± 0.0050	0.0702 ± 0.0103	0.0678 ± 0.0035	0.1399 ± 0.0140 ^a^
body weight (g)	23.13 ± 0.43	23.70 ± 0.45	24.04 ± 0.57	24.91 ± 0.55	21.77 ± 0.51	22.49 ± 0.58
spleen/body-ratio (g × g^−1^)	0.0028 ± 0.0001	0.0045 ± 0.0005 ^a^	0.0025 ± 0.0002	0.0028 ± 0.0004	0.0031 ± 0.0002	0.0062 ± 0.0007 ^a^

Data are presented as mean ± SEM. ^a^ *p* < 0.001, significantly different from IRAG1-WT.

**Table 2:** Hematological parameters of IRAG1-WT and IRAG1-KO mice.

Parameter	Total		Male		Female	
	*IRAG1-WT*	*IRAG1-KO*	*IRAG1-WT*	*IRAG1-KO*	*IRAG1-WT*	*IRAG1-KO*
HCT (%)	49.9 ± 0.9	45.1 ± 1.4 ^b^	49.5 ± 1.0	46.9 ± 1.7	50.3 ± 1.5	43.3 ± 2.2 ^a^
RBC (10^6^ × µL^−1^)	9.27 ± 0.30	7.28 ± 0.41 ^c^	9.60 ± 0.46	8.58 ± 0.47	8.92 ± 0.38	5.90 ± 0.43 ^c^
Hb (g × dL^−1^)	17.6 ± 0.2	14.5 ± 0.6 ^c^	17.9 ± 0.3	15.5 ± 0.6^c^	17.2 ± 0.3	13.2 ± 1.0 ^c^
MCH (fL)	55.7 ± 2.1	65.8 ± 3.0	53.0 ± 2.2	56.6 ± 3.4	58.6 ± 3.7	75.7 ± 3.6 ^a^
MCV (pg)	18.8 ± 0.6	19.1 ± 0.8 ^b^	19.2 ± 4.0	17.5 ± 0.5	18.2 ± 0.6	21.3 ± 1.1 ^b^
WBC (µL^−1^)	1807 ± 189	1811 ± 199	1617 ± 393	2600 ± 582	1933 ± 188	1586 ± 162

Data are presented as mean ± SEM. ^a^ *p* < 0.05, significantly different from IRAG1-WT; ^b^ *p* < 0.01, significantly different from IRAG1-WT; ^c^ *p* < 0.001, significantly different from IRAG1-WT.

## Data Availability

The datasets for this manuscript are not publicly available because the raw data supporting the conclusions of this manuscript will be made available by the authors, without undue reservation, to any qualified researcher. Requests to access the datasets should be directed to jens.schlossmann@chemie.uni-regensburg.de.

## Supplementary Materials

The following are available online at https://www.mdpi.com/article/10.3390/ijms22115458/s1, Figure S1: Haemoccult^®^ Test and analysis of spleen and body weight of six- and seven-week-old IRAG1-WT and IRAG1-KO mice, respectively, Figure S2: Hematological parameters of seven-week-old IRAG1-WT and IRAG1-KO mice, Figure S3: Hematological parameters of IRAG1-WT and IRAG1-KO mice (each 54–62 weeks), Figure S4: Reticulocytes production index (RPI) of IRAG1-WT and IRAG1-KO mice (each 54–62 weeks), Figure S5: Total protein images of spleens (A,B) and livers (C,D) of FLC expression, Figure S6: Total protein images of colons of the expression of several cGMP/PKGI signaling proteins, Figure S7: Total protein images of spleens of the expression of several cGMP/PKGI signaling proteins, Figure S8: Total protein images of stomachs of the expression of several cGMP/PKGI signaling proteins, Figure S9: Histological examination of the duodenum of IRAG1-WT and IRAG1-KO mice, Figure S10: Folate levels of IRAG1-WT and IRAG1-KO mice, Table S1: Distribution of sexes of the IRAG1-KO breed, Table S2: Distribution of genotypes of the IRAG1-KO breed, Table S3: Sequences of used primers in qRT-PCR.
