# Supplementary material for: Loss of PKGIβ/IRAG1 Signaling Causes Anemia-Associated Splenomegaly
Source: Int J Mol Sci. 2021 May 21;22(11):5458. doi: 10.3390/ijms22115458 (PMC8196906; doi:10.3390/ijms22115458)
Supplement: Supplementary file 1 [file ijms-22-05458-s001.zip › ijms-1237540-supplementary.pdf]

Supplement

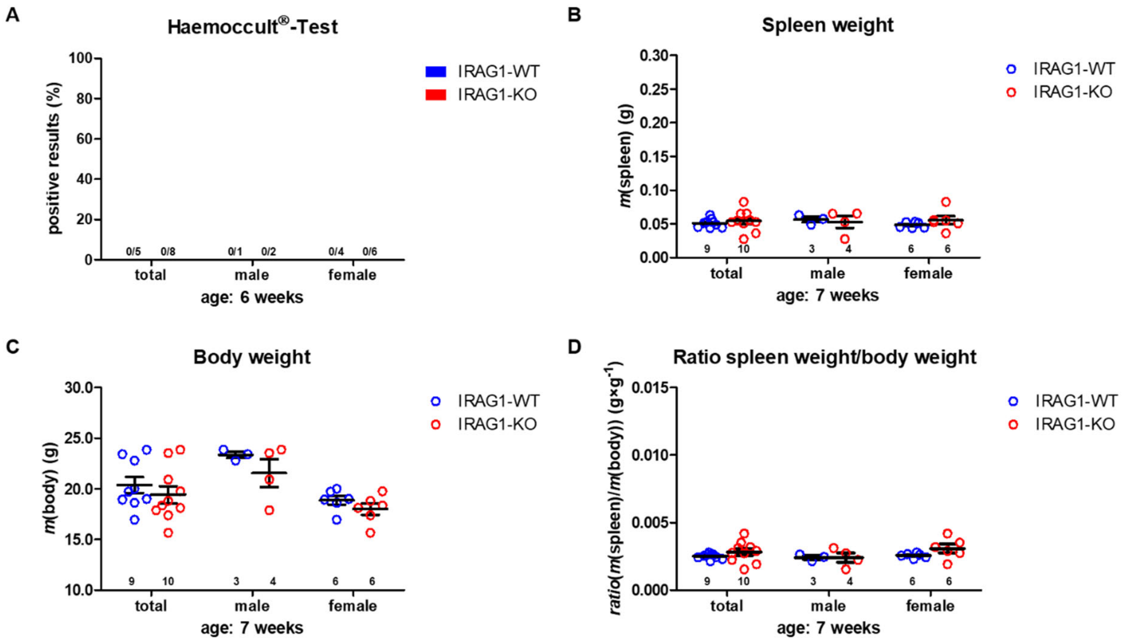

Figure S1. Haemoccult® Test and analysis of spleen and body weight of six- and seven-week-old IRAG1-WT and IRAG1-KO mice. (**A**) Haemoccult® Test. No significant differences were found in spleen weight (**B**), body weight (**C**) and spleen weight to body weight ratio (**D**). Circles indicate the individual value of each mouse and mean ± SEM is shown by bars and numbers of mice are shown in the graphs.

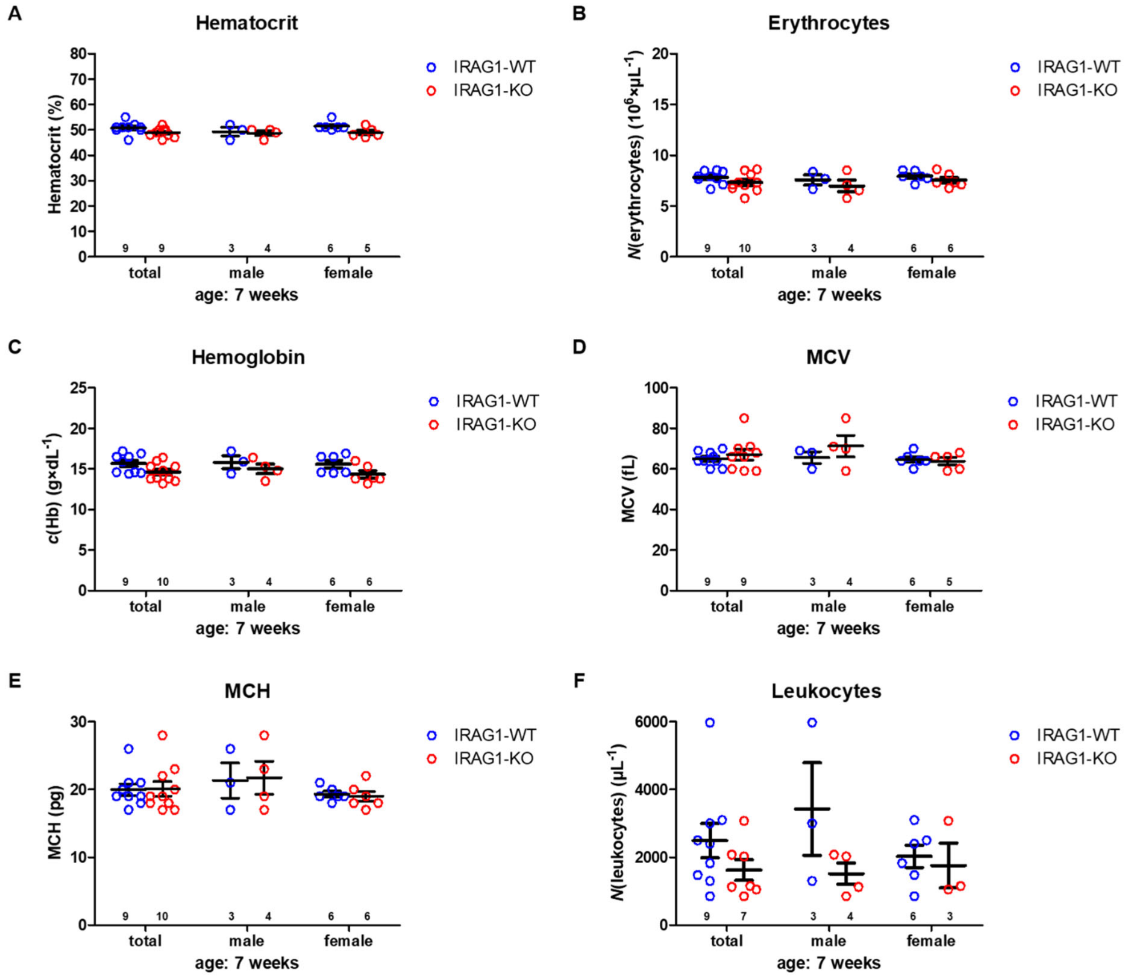

Figure S2. Hematological parameters of seven-week-old IRAG1-WT and IRAG1-KO mice. There were no differences between IRAG1-WT and IRAG1-KO mice in hematocrit (**A**), numbers of erythrocytes (**B**), hemoglobin (**C**), MCV (**D**), MCH (**E**) and leukocytes (**F**). Circles indicate the individual value of each mouse and mean ± SEM is shown by bars and numbers of mice are shown in the graphs.

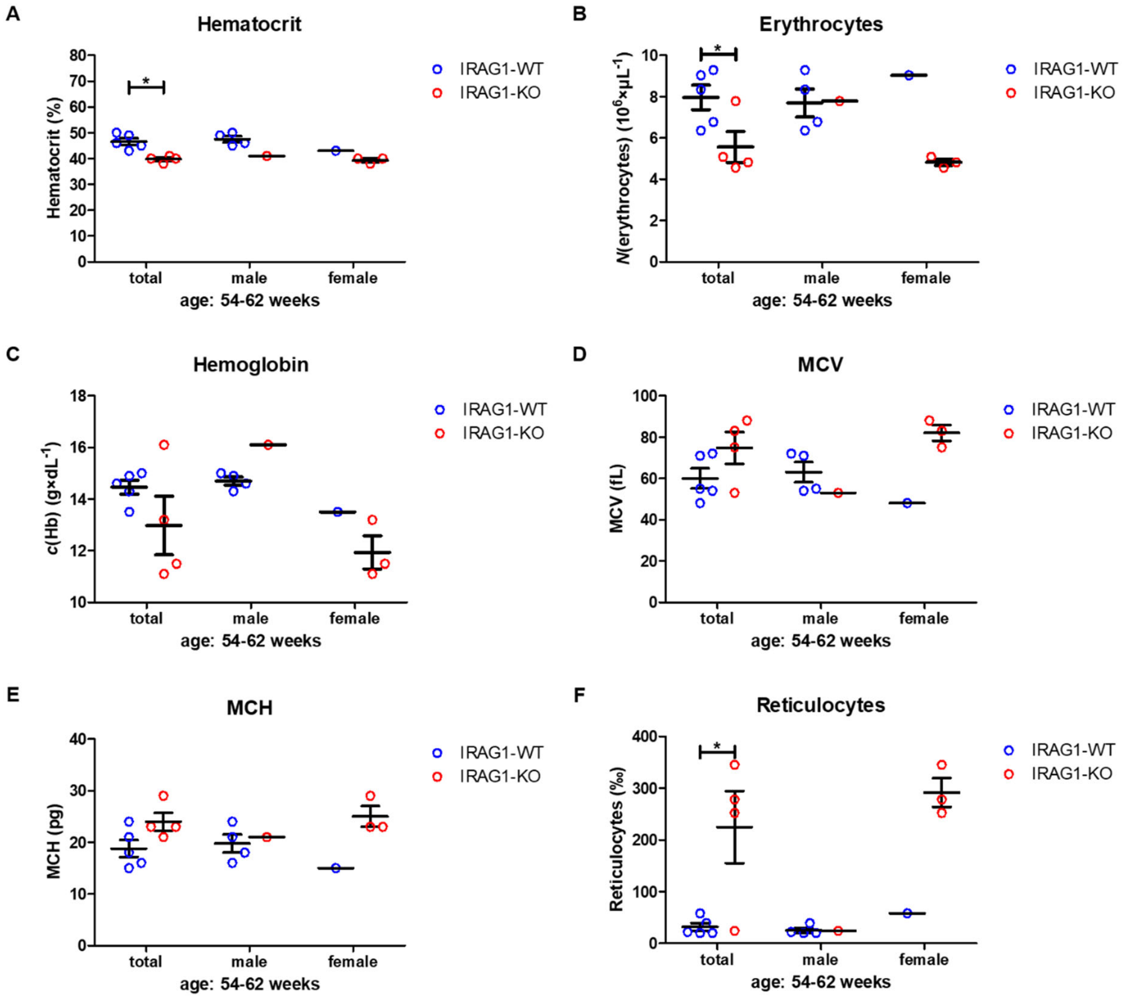

Figure S3. Hematological parameters of IRAG1-WT and IRAG1-KO mice (each 54-62 weeks). Hematocrit (**A**), numbers of erythrocytes (**B**), hemoglobin (**C**), MCV (**D**), MCH (**E**) and reticulocytes (**F**) of IRAG1-WT (total: n = 5; male: n = 4; female: n =1) and IRAG1-KO mice (total: n = 4; male: n =1; female: n = 3). Circles indicate the individual value of each mouse and mean ± SEM is shown by bars. Significant differences are shown by (*) ($p < 0.05$). For the analysis of the reticulocytes blood was taken as described in 4.3. 100 µl blood were mixed with 100 µl Brilliant cresyl blue solution (Merck KGaA, Darmstadt, Germany), incubated and a blood smear as done. Reticulocytes were counted from a total of 1000 cells under the microscope [1]. To reduce errors three blood smears of each mouse were analyzed and the of this three analyses were calculated. The investiator did not know the genotypes of the mice during the analysis

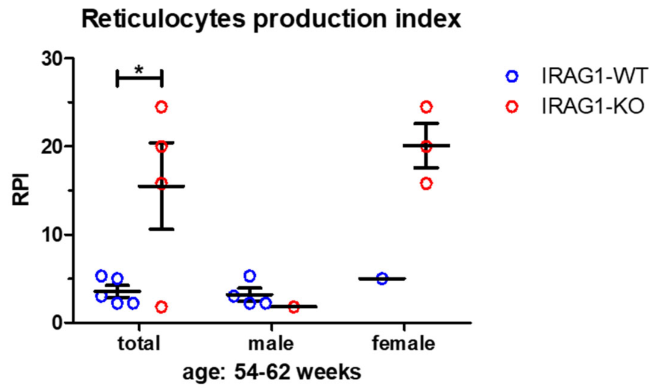

Figure S4. Reticulocytes production index (RPI) of IRAG1-WT and IRAG1-KO mice (each 54-62 weeks). RPIs of IRAG1-WT (total: n = 5; male: n = 4; female: n =1) and IRAG1-KO mice (total: n = 4; male: n =1; female: n = 3) were calculated with the data of Figure S2 [2]. Circles indicate the individual value of each mouse and mean ± SEM is shown by bars. Significant differences are shown by (*) ($p < 0.05$).

The RPI is a measurement for the assessment of the type of anemia and the results indicate that IRAG1-KO mice have an adequate regeneration of the blood.

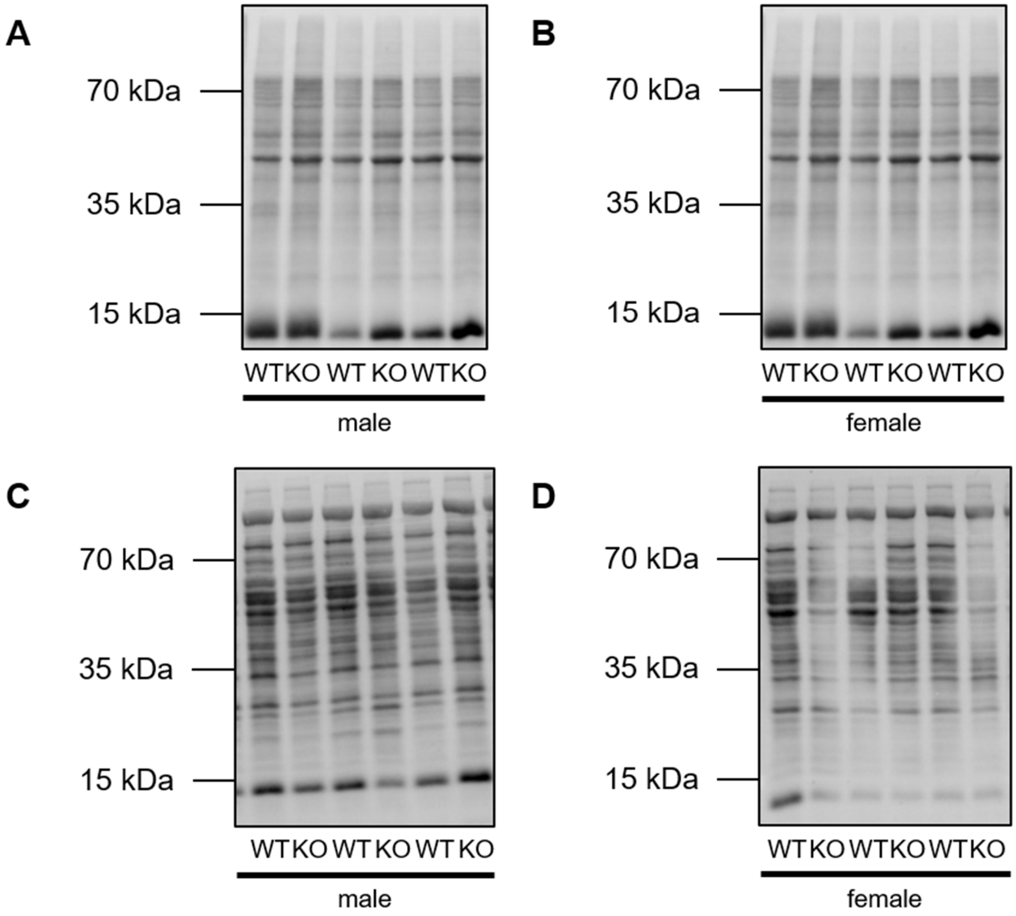

Figure S5. Total protein images of spleens (A, B) and livers (C, D) of FLC expression. Images of the total protein of western blots, which were used for the analysis of FLC expression (Figure 4A, C).

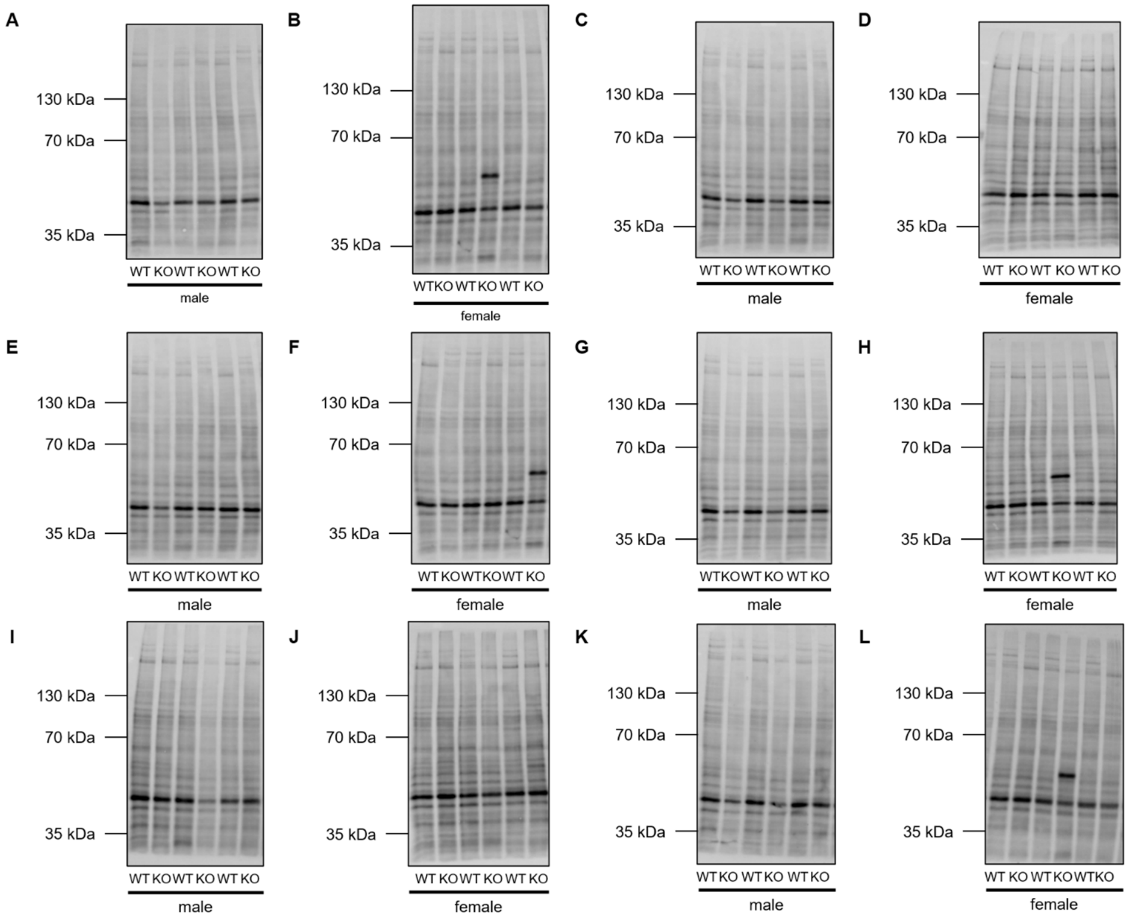

Figure S6. Total protein images of colons of the expression of several cGMP/PKGI signaling proteins. Images of the total protein of western blots, which were used for the analysis of cGMP/PKGI signaling proteins (Figure 5). (**A**, **B**): total protein of IRAG1 expression (Figure 5A). (**C**, **D**): total protein of IP_3R-I expression (Figure 5B). (**E**, **F**): total protein of PKGIβ expression (Figure 5C). (**G**, **H**): total protein of PKGIα expression (Figure 5D). (**I**, **J**): total protein of IP_3R-III expression (Figure 5E). (**K**, **L**): total protein of NO-GC-β1 expression (Figure 5F).

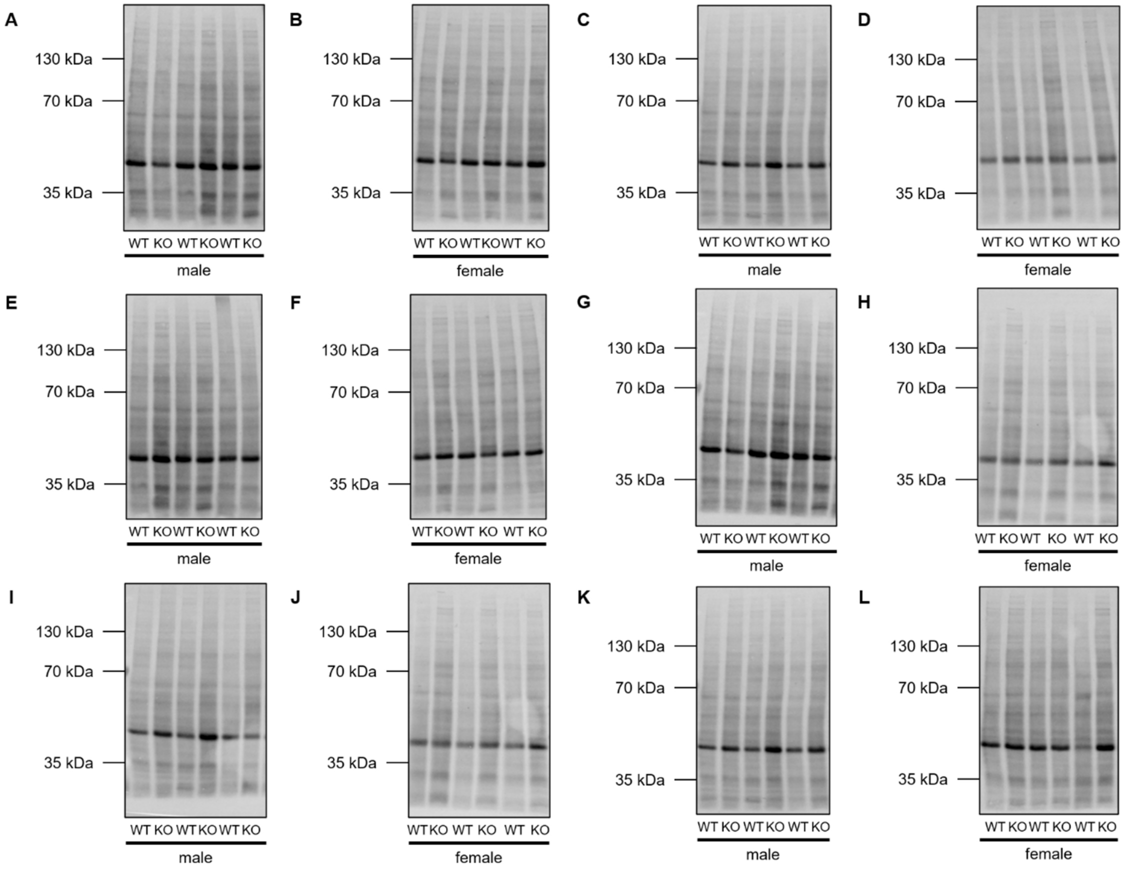

Figure S7. Total protein images of spleens of the expression of several cGMP/PKGI signaling proteins. Images of the total protein of western blots, which were used for the analysis of cGMP/PKGI signaling proteins (Figure 6). (**A**, **B**): total protein of IRAG1 expression (Figure 6A). (**C**, **D**): total protein of IP_3R-I expression (Figure 6B). (**E**, **F**): total protein of PKGIβ expression (Figure 6C). (**G**, **H**): total protein of PKGIα expression (Figure 6D). (**I**, **J**): total protein of IP_3R-III expression (Figure 6E). (**K**, **L**): total protein of NO-GC-β1 expression (Figure 6F).

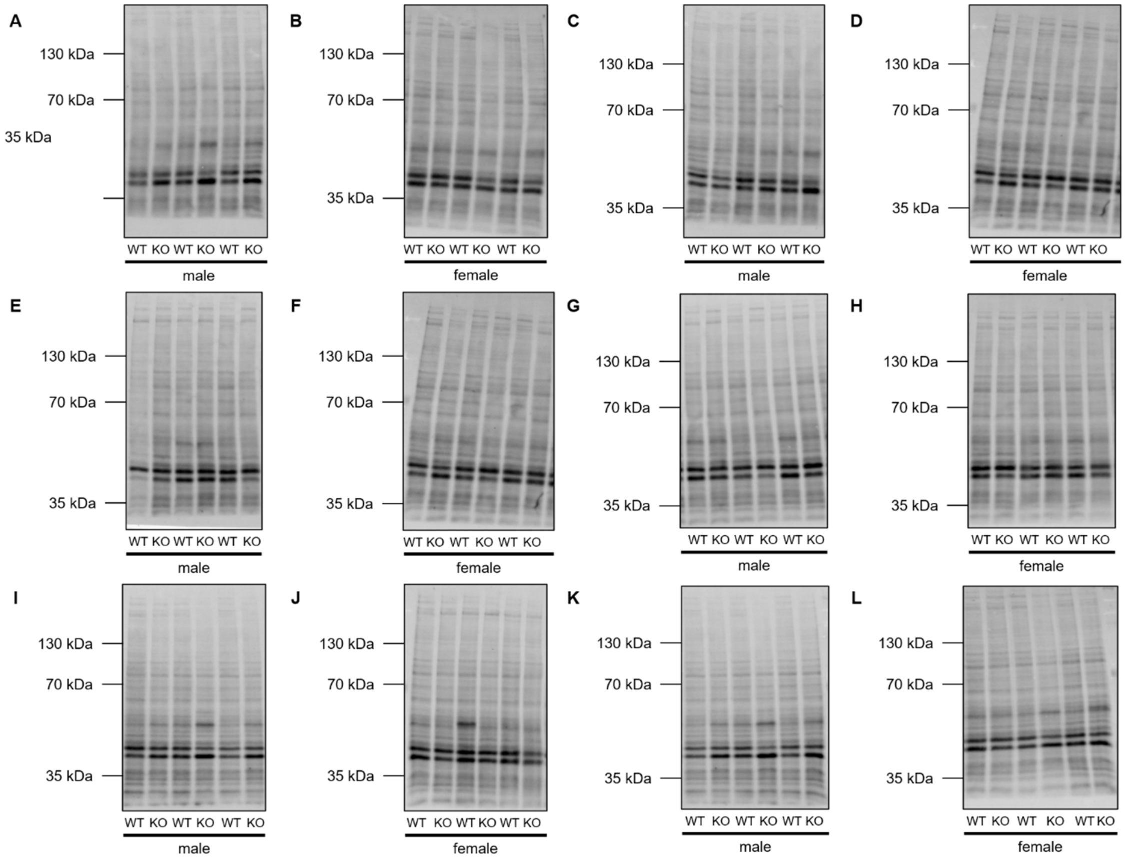

Figure S8. Total protein images of stomachs of the expression of several cGMP/PKGI signaling proteins. Images of the total protein of western blots, which were used for the analysis of cGMP/PKGI signaling proteins (Figure 7). (**A**, **B**): total protein of IRAG1 expression (Figure 7A). (**C**, **D**): total protein of IP_3R-I expression (Figure 7B). (**E**, **F**): total protein of PKGIβ expression (Figure 7C). (**G**, **H**): total protein of PKGIα expression (Figure 7D). (**I**, **J**): total protein of IP_3R-III expression (Figure 7E). (**K**, **L**): total protein of NO-GC-β1 expression (Figure 7F).

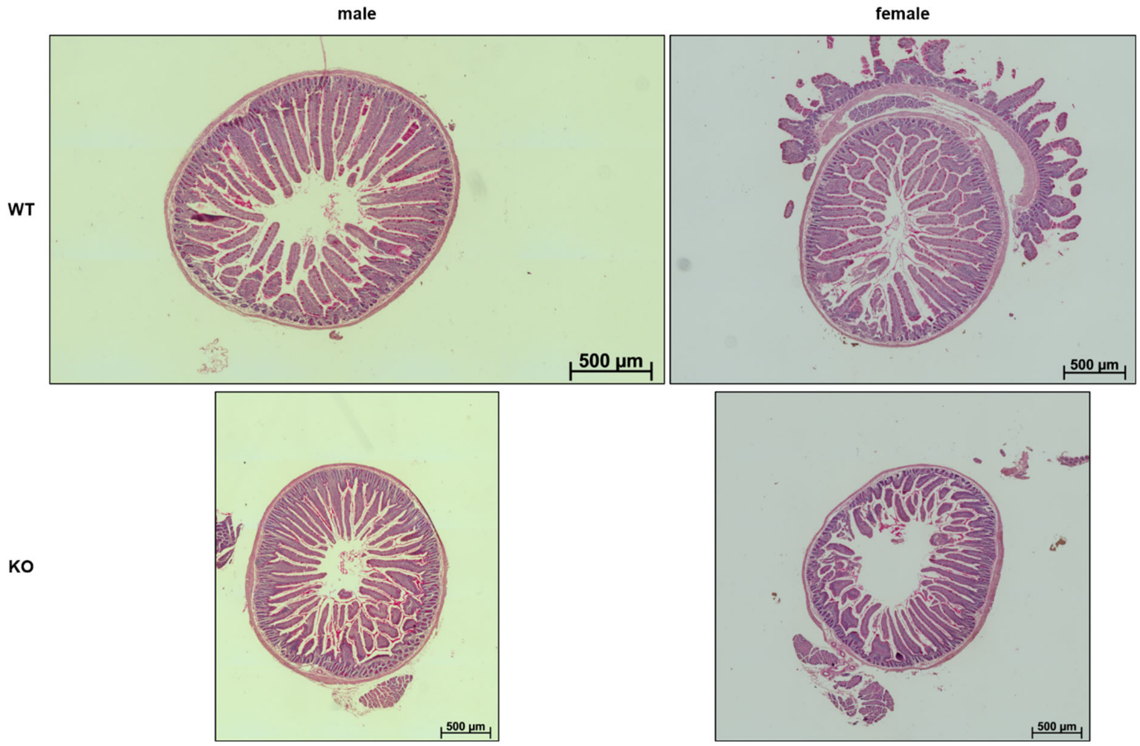

Figure S9. Histological examination of the duodenum of IRAG1-WT and IRAG1-KO mice. Duodena 15 weeks old male and female IRAG1-WT and IRAG1-KO mice were prepared and sectioned as described in 4.8. Periodic acid Schiff (PAS) staining was done in accordance to the standard protocol of the PAS staining kit (HP01.1, Carl Roth® GmbH, Karlsruhe, Germany).

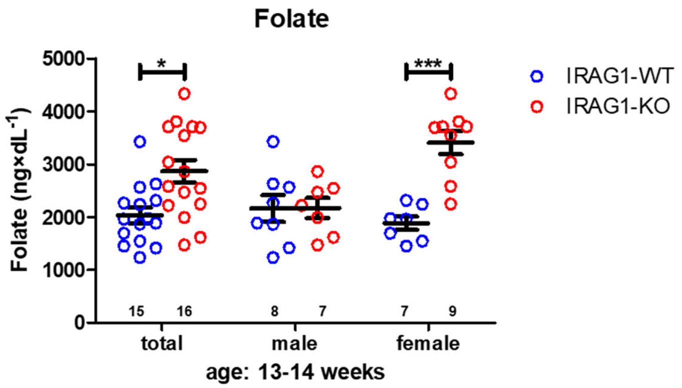

Figure S10. Folate levels of IRAG1-WT and IRAG1-KO mice. Folate levels of male and female 13-14 weeks old IRAG1-WT and IRAG1-KO mice were determined via ELISA. No differences were found between male IRAG1-WT and IRAG1-KO mice, whereas in female and in the total analysis folate levels of IRAG1-KO mice were significantly increased. Circles indicate the individual value of each mouse and mean ± SEM is shown by bars. Significant differences are shown by (*) ($p < 0.05$), (***) ($p < 0.001$).

ELISA of folate was performed of heparinized plasma samples of 13-14 weeks old male and female IRAG1-WT and IRAG1-KO mice. Therefore, we used the Folic Acid (FA) ELISA kit (ABIN6966851) of antibodies-online GmbH (Aachen, Germany).

Table S1. Distribution of sexes of the IRAG1-KO breed.

Sexes of young mice (4-weeks-old), which were bred out of heterozygous mating (genotype of parent animals: *Irag1*$^{+/-}$), were analysed after separation from the parent animals. The data represent all possible three genotypes.

	sex	
	male	female
**number**	164	161
**[%]**	50.46	49.54

Table S2. Distribution of genotypes of the IRAG1-KO breed.

Evaluation of the genotype distribution of heterozygous mating (genotype of parent animals: *Irag1*$^{+/-}$) after separation from the parent animals.

	genotype		
	Irag1$^{+/+}$	*Irag1*$^{-/-}$	*Irag1*$^{+/-}$
**number**	81	81	163
**[%]**	24.92	24.92	50.16

Table S3. Sequences of used primers in qRT-PCR.

gene	fwd.	rev.
Gapdh	5'-CACCAGGGCTGCCATTTGCA-3'	5'-GCTCCACCCTTCAAGTGG-3'
Gucy1b1	5'-TGCAAGCAAAGTCCTCAACCT-3'	5'-ATCCCAGGACACGCAAGATG-3'
Hamp	5'-TGAGCAGCACCACCTATCTC-3'	5'-ACAGCAGAAGATGCAGATGG-3'
Irag1	5'-GAGGCTTTCCCACCGACAC-3'	5'-TTGGAGAAACAGCTGATGGGG-3'
Itpr1	5'-CCACAGAGCAGGAGCTTGAA-3'	5'-GTGACACTGAACCTCAGCCA-3'
Itpr3	5'-CTGGATCAGGACTGGTCAGC-3'	5'-GAGGACACGCGGCCTTT-3'
Prkg1a	5'-CGCCAGGCGTTCCGGAAGTT-3'	5'-GTGCAGAGCTTCACGCCTT-3'
Prkg1b	5'-TGGACAAGTATCGCTCGGTG-3'	5'-GATCCTTCGACTGTGGGCTC-3'
Tfr1	5'-TACCTGGGCTATTGTAAGCG-3'	5'-TTTGAGATCCAGCCTCACG-3'

References

1. Rick, W. *Klinische Chemie und Mikroskopie*, Sechste, überarbeitete und erweiterte Auflage; Springer: Berlin, Heidelberg, 1990, ISBN 9783642840388.
2. Nebe, C.T.; Diem, H.; Heimpel, H. Aktuelle Aspekte zur Bestimmung der Retikulozytenzahl / Current aspects of reticulocyte analysis. *LaboratoriumsMedizin* **2010**, *34*, 295–304, doi:10.1515/jlm.2010.057.
